# Physical and functional performance assessment in pediatric oncology: a systematic review

**DOI:** 10.1038/s41390-021-01523-5

**Published:** 2021-04-15

**Authors:** Regine Söntgerath, Julia Däggelmann, Sabine V. Kesting, Corina S. Rueegg, Torge-Christian Wittke, Simon Reich, Katharina G. Eckert, Sandra Stoessel, Carolina Chamorro-Viña, Joachim Wiskemann, Peter Wright, Anna Senn-Malashonak, Vanessa Oschwald, Anne-Marie Till, Miriam Götte

**Affiliations:** 1 Department of Pediatric Oncology, Hematology and Hemostaseology, University Hospital Leipzig, Leipzig, Germany; 2 Department of Molecular and Cellular Sports Medicine, Institute of Cardiology and Sports Medicine, German Sport University Cologne, Cologne, Germany; 3 Institute of Preventive Pediatrics, Department of Sport and Health Sciences, Technical University of Munich, Munich, Germany; 4 Kinderklinik München Schwabing, TUM School of Medicine, Department of Pediatrics and Children’s Cancer Research Center, Technical University of Munich, Munich, Germany; 5 Oslo Centre for Biostatistics and Epidemiology, Oslo University Hospital, Oslo, Norway; 6 Krukenberg Cancer Center, University Medicine Halle (Saale), Halle, Germany; 7 Working Group Exercise Oncology Division of Medical Oncology, University Clinic Heidelberg and National Centre for Tumor Diseases (NCT), Heidelberg, Germany; 8 Department of Health Management & Public Health, IST University of Applied Sciences Düsseldorf, Düsseldorf, Germany; 9 Center for Pediatric and Adolescent Medicine, Childhood Cancer Center, University Medical Center Mainz, Mainz, Germany; 10 Faculty of Kinesiology, Calgary, Alberta Canada; 11 Department of Sport, Health Sciences and Social Work, Oxford Brookes University, Oxford, UK; 12 Department of Pediatric Oncology, Hematology and Hemostaseology, Goethe University Clinic Frankfurt, Frankfurt am Main, Frankfurt, Germany; 13 Department of Pediatric Hematology and Oncology, University Hospital Schleswig-Holstein, Lübeck, Germany; 14 Department of Pediatric Hematology and Oncology, University Hospital Essen, Pediatrics III, Essen, Germany

## Abstract

**Background:**

Research indicates reduced physical performance from diagnosis into survivorship of pediatric cancer patients. However, there is no systematic information or guideline available on the methods to assess physical performance and function in this population. The purpose was to systematically compile and describe assessments of physical performance and function in patients and survivors of pediatric cancer, including cardiorespiratory fitness, muscle strength, speed, balance, flexibility, functional mobility, gait and motor performance test batteries.

**Methods:**

We searched the databases PubMed, SPORTDiscus, and Cochrane Database and performed abstract and full-text selection of 2619 articles according to the Cochrane Handbook of Systematic Reviews. Information on patients characteristics, assessments, information on validity and reliability, and relevant references was extracted.

**Results:**

In summary, 63 different assessments were found in 149 studies including 11639 participants. Most studies evaluated cardiorespiratory fitness and muscle strength with the majority conducted off treatment. Some outcomes (e.g. speed) and diagnoses (e.g. neuroblastoma) were severely underrepresented. With the exception of gait, leukemia patients represented the largest group of individuals tested.

**Conclusions:**

Insufficient data and patient heterogeneity complicate uniform recommendations for assessments. Our results support researchers and practitioners in selecting appropriate assessment to meet their specific research questions or individual daily practice needs.

**Impact:**

- This systematic review includes 149 studies and provides a comprehensive summary of 63 assessments to evaluate cardiorespiratory fitness, muscle strength, speed, balance, flexibility, functional mobility, gait or motor performance test batteries in patients and survivors of pediatric cancer.
- We present the most studied fields within the pediatric cancer population, which are cardiorespiratory fitness and muscle strength, off treatment phase, and leukemia patients.
- We propose research priorities by identification of subgroups in terms of cancer type, phase of treatment, and outcome of interest that are underrepresented in studies currently available.

## Introduction

Age-appropriate healthy physical and functional development of infants, children, and adolescents is an important prerequisite for participation in physical activity and sports representing a major determinant of a long-term active and healthy lifestyle.^1^ Physical and functional performance of children and adolescents during and after cancer treatment has been the interest of a growing number of studies during past decades. Current literature presents increasing evidence that childhood cancer patients and survivors are challenged by physical performance limitations such as reduced cardiorespiratory fitness, muscle strength, balance, gait, functional mobility, and flexibility/range of motion.^2–4^ Influencing factors for these impairments might be the cancer itself, side effects of medical therapy, and inactivity during and after treatment.^5^ Study results demonstrate reduced physical performance shortly after diagnosis,^6^ during acute treatment,^3^ and persisting throughout survivorship.^7^ This is specifically concerning as physical performance limitations are linked to an increased incidence of unemployment and low income.^8^

At the same time, preliminary exercise intervention studies provide promising results in terms of efficacy to improve physical performance and fitness.^9–11^ However, evaluation of those positive effects found in research interventions with childhood cancer populations is difficult due to the large number of different physical and functional performance assessments that have been used in pediatric oncology research. An overview of assessments could help future researchers when planning a study on exercise and fitness in child and adolescent cancer patients and survivors. Few attempts have been done to summarize and describe tests performed and used in this population. Grimshaw et al.^12^ summarized subjective and objective tools to measure physical function and physical activity in the age group 0–18 years with a focus on the evaluation of measurement properties. Another group of researchers^13^ listed evaluation tools used in childhood cancer physical activity/exercise studies or community-based programs that assess motor performance, physical literacy, well-being, quality of life (QoL), and health behavior, but assessments of physical performance and fitness were excluded. However, no review has predefined the categories of physical and functional performance relevant to health and exercise science^14^ in order to systematically search and summarize them. Thus, the aim of the present systematic review is to summarize in detail all assessments used to measure cardiorespiratory fitness, muscle strength, speed, balance, flexibility, functional mobility, gait, and motor performance in interventional and non-interventional studies with childhood cancer patients and survivors. This summary is intended to support researchers and therapists in selecting the most appropriate assessments for their individual purposes and needs.

## Materials and methods

This paper was written according to the Preferred Reporting Items for Systematic Reviews and Meta-Analyses (PRISMA Statement).^15^

### Data sources and searches

We systematically searched PubMed, Cochrane Central Register of Controlled Trials and SPORTDiscus from database inception to 13 February 2020. The search strategy (see Appendix 1) included Medical Subject Headings with terms and text words to identify studies conducted with children, adolescents, or adults during or after childhood cancer treatment who underwent any assessment for either physical or functional performance. In addition, references of relevant reviews and reference lists of included studies were screened. The specific outcomes of interest were assessments for cardiorespiratory fitness, muscle strength, speed, balance, flexibility, functional mobility, gait, and motor performance assessed in test batteries. Motor performance test batteries measuring physical performance provide an important overview of performance levels and motor development and are of great importance in children and adolescents. In pediatric oncology and chronically ill children, they are usually assessing performance of general motor skills. Data on validity and reliability of the included assessments in pediatric cancer patients and survivors were extracted from the included full texts and associated references.

### Study selection

After exclusion of duplicates, three teams of two researchers each independently reviewed titles and abstracts of the identified articles. Studies were excluded for the following reasons: (i) less than 75% of the population were diagnosed with cancer <21 years, (ii) the outcome was no measure of either physical or functional performance as defined above, (iii) any non-original articles (e.g. reviews, congress abstracts, commentaries or letters without data), (iv) duplicates that were not identified as such before, (v) studies without description of assessment used (vi) studies/assessment with less than five participants, or (vii) full-texts that were not available in English or German. We included all types of studies and had no restriction in terms of publication date. In case of disagreement between the two reviewers, articles were discussed between these two and if no consensus could be reached, a third reviewer was consulted. After final inclusion of abstracts, the respective full texts were reviewed independently as described above.

### Data extraction, synthesis, and analysis

Relevant data from the included full texts were extracted and organized into standardized data tables. During the data extraction process, the following information was extracted from all texts: study citation, characteristics of the study population (sample sizes, age ranges, diagnoses, stage of cancer treatment), assessments used and their measurement properties, and relevant references for further information. In terms of measurement properties, information regarding validity and reliability of the assessment was only extracted if those were evaluated in the childhood cancer study sample. Based on these tables, assessments were sorted into predefined health-related and skill-related categories as defined by the American College of Sports Medicine^14^ (cardiorespiratory fitness, muscle strength, speed, balance, flexibility). In addition to these main motor domains, functional mobility, gait, and motor performance test batteries which have been identified to be of high relevance for the population of children with cancer^3,16,17^ and for coping with everyday life and participation with peers^18^ were included. For each single assessment (e.g. 6-minute walk test (6MWT) in the category cardiorespiratory fitness) all information about the study participants was merged from the studies using this particular assessment. Diagnoses were grouped into categories, i.e. leukemia/lymphoma (as hematological tumors), bone tumor, CNS tumors, and others. This classification was made because individuals after bone tumors or CNS tumors are known to suffer from more severe motor deficits due to the underlying disease.^19^ In case of insufficient information, study authors were contacted via email. If no answer was received, information was taken from the manuscript as specific as possible.

## Results

### Literature search results

**Fig. 1:**
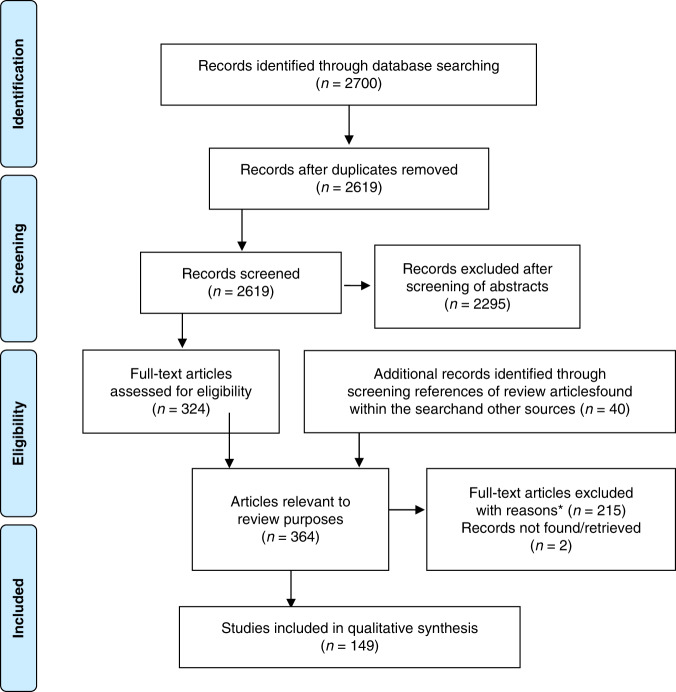
PRISMA flow diagram showing the reference selection process. *Reasons for exclusion: less than 75% of the population were diagnosed with cancer measure of either physical or functional performance (*n* = 55), any non-original articles (e.g. reviews, congress abstracts, commentaries or letters without data) (*n* = 31), duplicates that were not identified as such before (*n* = 10), (v) studies without description of assessment used (*n* =9), (vi) studies/assessment with less than five participants (*n* = 11), or (vii) full-texts that were not available in English or German (*n* = 13).

Figure 1 displays the flow of studies through the review process. After 81 duplicates were removed, 2619 records underwent abstract screening, of which 2295 were excluded and 324 articles were retained for full-text review. Additional 40 articles, identified through reference list screening of reviews and other sources, were then added to full-test screening, resulting in 364 articles. Of those, 215 articles were excluded with reasons and 149 full texts^3,6,10,20–165^ (see Appendix 2 for detailed study information) were included for data extraction, representing 5.7% of screened abstracts and 40.7% of screened full text articles. Agreement between the reviewers for abstract screening ranged between 82 and 96% and for the full text screening between 84 and 93%.

### Study characteristics

**Fig. 2:**
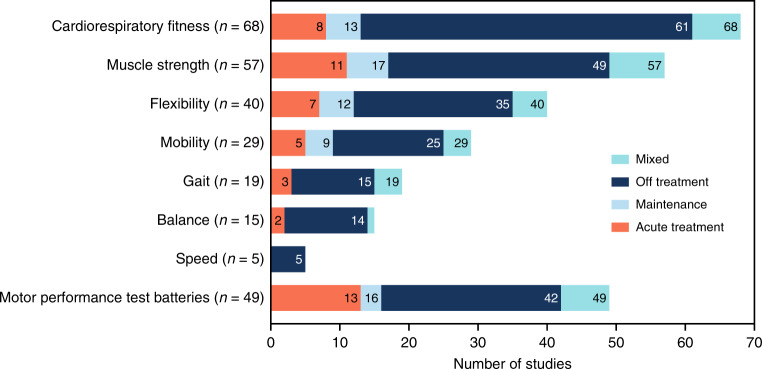
Number of studies for each outcome measure, indicating the phase of therapy in which the studies were conducted. Please note: The sum of assessments in Fig. 2 (*n* = 282) is greater than the number of all included studies (*n* = 149), because in many studies assessments from several categories were included.

**Fig. 3:**
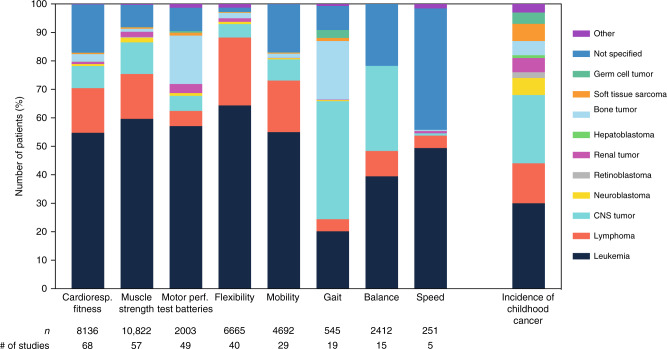
Distribution of the types of cancer (%) that were included in the assessments of the eight categories of physical performance and function. The bar on the far right shows the incidence of childhood cancer as a reference. Note: Number of persons tested specified here (e.g. *n* = 8136 for cardiorespiratory fitness) differs from number of study participants (*n* = 7936, see section on cardiorespiratory fitness above), because some study participants were tested using several test methods.

In summary, all 149 studies, describing 63 different assessment methods, included in this systematic review were published between 1984 and 2020. Of those, *n* = 1 study was published between 1984 and 1990, *n* = 18 studies between 1991 and 2000, *n* = 48 studies between 2001 and 2010, and *n* = 82 studies between 2011 and 2020. The studies included a total number of *n* = 11,639 participants being treated for childhood cancer and/or having received hematopoietic stem cell transplantation (HSCT). Of these, *n* = 6295 (54.1%) were diagnosed with leukemia, *n* = 1408 (12.1%) with lymphoma, *n* = 1271 (10.9%) with tumors of the central nervous system (CNS), *n* = 76 (0.7%) with neuroblastoma, *n* = 12 (0.1%) with retinoblastoma, *n* = 149 (1.3%) with renal tumor, *n* = 3 (0.03%) with hepatoblastoma, *n* = 692 (5.9%) with bone tumor, *n* = 68 (0.6%) with soft tissue sarcoma, *n* = 34 (0.3%) with germ cell tumor, and *n* = 56 (0.5%) with other malignancies. In *n* = 1575 cases (13.5%) a classification was not possible due to a missing detailed description in the full texts. While 22 studies (14.8%) took place during active cancer treatment, 9 studies (6.0%) were conducted during maintenance therapy and 99 studies (66.4%) after treatment. Nineteen studies (12.8%) included participants during different phases of medical treatment. The age of participants ranged between 1.0 and 68.3 years. Most studies analyzed a parameter of cardiorespiratory fitness, followed by strength, motor performance in test batteries, flexibility, functional mobility, gait and balance. Only five studies evaluated speed (Fig. 2). Considering the incidence of childhood cancer,^166^ the number of individuals tested in the categories of physical performance and function deviates from the incidence of the tumor type. An overview of the distribution of diagnoses within each category and overall childhood cancer incidence rates are presented in Fig. 3. Some physical performance categories, like cardiorespiratory fitness and muscle strength, were tested in many different types of cancer. However, in all categories, with the exception of gait, individuals with leukemia were over-represented. For gait and motor performance test batteries, the inclusion of bone tumor patients was far above the percentage incidence of bone tumors, whereas in the other six categories, bone tumors as well as other solid tumors were investigated less frequently.

### Results on methods to assess physical and functional performance

In total, 63 different assessments were used to evaluate at least one of the eight categories of physical performance and/or function. Between 2 and 16 different assessments were used to evaluate one of the eight categories. The largest heterogeneity in assessment type, calculated as the number of assessment types divided by the number of studies, was in gait with 8 different assessments from 19 studies (0.42), balance with 6 different assessments from 15 studies, and speed with 2 different assessments from 5 studies (0.40). To assess motor performance with test batteries, 16 different test batteries were used in 49 studies (0.33). Strength was evaluated with 16 different measures in 57 studies (0.28) and mobility with 5 assessments in 29 studies (0.17). The greatest homogeneity in measurement techniques was for cardiorespiratory fitness (0.10) and flexibility (0.08) (7 and 3 different methods in 68 and 40 studies, respectively). The different methods are summarized in Tables 1–8 with more details in Appendices 3–10.

**Table 1:** Summary of study methods assessing cardiorespiratory fitness in pediatric oncology.

Assessment	No. of studies	Total sample size^a^	Type of cancer				Age in years (range)	Phases of treatment			Validity^c^	Reliability^c^
			Leukemia/lymphoma	Bone tumor	CNS tumor	Other^b^		During	Maint.	Off		
Maximal CPET	33	1170	✓	✓	✓	✓	3.5–41	✓	✓	✓	–	–
6MWT	26	6180	✓	✓	✓	✓	3.5–63.8	✓	✓	✓	–	–
Submaximal CPET	5	457	✓	–	✓	✓	7–44.6	–	–	✓	–	–
9MWT	4	154	✓	✓	–	✓	4–27	–	✓	✓	✓	✓
Wingate anaerobic test	3	58	✓	–	–	✓	7.7–23.8	–	–	✓	–	–
2MWT	2	91	✓	–	✓	✓	6–45	–	✓	✓	–	–
PACER	2	25	✓	–	✓	–	4–18	–	✓	✓	–	–

*2MWT* 2-minute walk test, *6MWT* 6-minute walk test, *9MWT* 9-minute walk test, *CPET* cardiopulmonary exercise test, *maint.* maintenance treatment, *No.* number, *PACER* progressive aerobic cardiovascular endurance run.

^a^Only study participants who performed the assessments were counted.

^b^Including other cancer diagnoses and diagnoses that were not clearly specified.

^c^If evaluated in a childhood cancer population.

**Table 2:** Summary of study methods assessing muscle strength in pediatric oncology.

Assessment	No. of studies	Total sample size^a^	Type of cancer				Age in years (range)	Phase of treatment			Validity^c^	Reliability^c^
			Leukemia/ lymphoma	Bone tumor	CNS tumor	Other^b^		During	Maint.	Off		
Grip strength test	27	4451	✓	✓	✓	✓	3.5–64	✓	✓	✓	–	–
Hand held dynamometry	17	830	✓	✓	✓	✓	4–58	✓	✓	✓	–	✓
Isokinetic fynamometry	10	3718	✓	✓	✓	✓	10.5–64	–	✓	✓	–	✓
Sit-up test	10	339	✓	✓	✓	✓	5–62.2	✓	✓	✓	–	–
Push-up test	6	239	✓	✓	✓	✓	4–62.2	–	✓	✓	–	–
Manual muscle test	6	165	✓	✓	-	✓	2–50+	✓	✓	✓	–	–
Repetition maximum	6	104	✓	✓	✓	✓	4–23	✓	✓	✓	–	✓
Repeated squatting	5	182	✓	–	✓	✓	6–30	–	–	✓	–	–
Back extension test	4	220	✓	–	–	✓	6–62.2	–	–	✓	–	–
Isometric dynamometry	3	143	✓	–	–	✓	5–30	✓	–	✓	–	–
Chair-stand test	3	100	✓	–	✓	✓	3.5–18	✓	–	✓	–	–
Leg lift test	2	128	✓	–	–	–	6–30	–	–	✓	–	–
Vertical jump	2	92	✓	–	–	✓	16–62.2	–	–	✓	–	–
Shoulder lift test	1	21	✓	–	–	–	16–30	–	–	✓	–	–
Standing broad jump	1	18	✓	–	–	–	7.55 ± 2.43^d^	–	–	✓	–	–
Lateral step test	1	12	✓	–	✓	–	6–18	–	–	✓	–	–

*maint*. maintenance treatment, *No*. number.

^a^Only study participants who performed the assessments were counted.

^b^Including other cancer diagnoses and diagnoses that were not clearly specified.

^c^If evaluated in a childhood cancer population.

^d^No information on minimum/maximum.

**Table 3:** Summary of study methods assessing running speed in pediatric oncology.

Assessment	No. of studies	Total sample size^a^	Type of cancer				Age in years (range)	Phases of treatment			Validity^c^	Reliability^c^
			Leukemia/lymphoma	Bone tumor	CNS tumor	Other^b^		During	Maint.	Off		
Shuttle run	3	146	✓	–	–	✓	6–30	–	–	✓	–	✓
60 m run	2	105	✓	✓	✓	✓	11 ± 3^d^	–	–	✓	–	–

*maint.* maintenance treatment, *No.* number.

^a^Only study participants who performed the assessments were counted.

^b^Including other cancer diagnoses and diagnoses that were not clearly specified.

^c^If evaluated in a childhood cancer population.

^d^No information on minimum/maximum.

**Table 4:** Summary of study methods assessing balance in pediatric oncology.

Assessment	No. of studies	Total sample size^a^	Type of cancer				Age in years (range)	Phases of treatment			Validity^c^	Reliability^c^
			Leukemia/lymphoma	Bone tumor	CNS tumor	Other^b^		Active	Maint.	Off		
**Posturography**												
SOT on dynamic posturography system (various):	7	1805	✓	–	✓	✓	10–63.8	–	–	✓	–	–
Balance tests (various) on force platforms	4	292	✓	✓	✓	✓	4–25.2	–	–	✓^d^	–	–
Ultrasound-based motion analysis of postural sway	1	22	–	–	✓	–	11–39	–	–	✓	–	–
**Non-posturography**												
The Berg balance test	1	156	–	–	✓	–	18–58	–	–	✓	–	–
Flamingo balance test	1	75	✓	–	✓	✓	11.3 ± 3.1^e^	✓	–	–	–	–
Single leg stance	1	62	✓	–	–	–	1–22	✓	–	–	–	–

*maint*. maintenance treatment, *No.* number, *SOT* sensory organization test.

^a^Only study participants who performed the assessments were counted.

^b^Including other cancer diagnoses and diagnoses that were not clearly specified.

^c^If evaluated in a childhood cancer population.

^d^One study was performed during inpatient rehabilitation potentially including patients still receiving maintenance treatment.

^e^No information on minimum/maximum.

**Table 5:** Summary of study methods assessing flexibility in pediatric oncology.

Assessment	No. of studies	Total sample size^a^	Type of cancer				Age in years (range)	Phases of treatment			Validity^c^	Reliability^c^
			Leukemia/lymphoma	Bone tumor	CNS tumor	Other^b^		During	Maint.	Off		
Goniometry	33	3764	✓	✓	✓	✓	1–64	✓	✓	✓	–	✓
Sit and reach	12	2830	✓	✓	✓	✓	4–64	✓	✓	✓	–	–
Side-bending	1	71	✓	–	–	✓	18.8–62.2	–	–	✓	–	–

*maint*. maintenance treatment, *No.* number.

^a^Only study participants who performed the assessments were counted.

^b^Including other cancer diagnoses and diagnoses that were not clearly specified.

^c^If evaluated in a childhood cancer population.

**Table 6:** Summary of study methods assessing functional mobility in pediatric oncology.

Assessment	No. of studies	Total sample size^a^	Type of cancer				Age in years (range)	Phases of treatment			Validity^c^	Reliability^c^
			Leukemia/lymphoma	Bone tumors	CNS tumors	Other^b^		active	Maint.	Off		
TUG 3 m	25	4,283	✓	✓	✓	✓	3.5–64	✓	✓	✓	✓	✓
TUDS	13	314	✓	✓	–	✓	3.5–27	✓	✓	✓	–	✓
TUG 10 m	3	22	✓	–	–	–	4–16	–	✓	✓	–	✓
Floor to stand performance	1	62	✓	–	–	–	1–22	✓	*–*	*–*	–	–
Stand up from bed rest exam	1	11	✓	–	–	✓	3.5–15	✓	*–*	*–*	–	–

*maint*. maintenance treatment, *No.* number, *TUDS* timed up and down stairs test, *TUG* timed up and go test.

^a^Only study participants who performed the assessments were counted.

^b^Including other cancer diagnoses and diagnoses that were not clearly specified.

^c^If evaluated in a childhood cancer population.

**Table 7:** Summary of study methods assessing gait in pediatric oncology.

Assessment	No. of studies	Total sample size^a^	Type of cancer				Age in years (range)	Phases of treatment			Validity^c^	Reliability^c^
			Leukemia/lymphoma	Bone tumor	CNS tumor	Other^b^		During	Maint.	Off		
Video-recording and force platforms	6	111	–	✓	✓	✓	3–35	–	–	✓^d^	–	–
Video-recording	4	210	–	✓	✓	✓	4–24	–	✓	✓^e^	–	–
Video-recording, force plates and EMG	3	67	✓	✓	–	✓	5–68.3	–	✓	✓	–	–
GAITRite	2	58	✓	–	–	✓	5–22	✓	–	–	–	–
Visual observation	1	62	✓	–	–	–	1–22	✓	–	–	–	–
10 m walk test	1	16	✓	–	✓	✓	6–19	–	✓	✓	–	–
Microgate optogait 2D Gait analysis system	1	13	✓	–	✓	✓	6–15.8	–	–	✓	–	–
EMG analysis (treadmill)	1	8	–	✓	–	–	N/A^f^	–	–	✓	–	–

*EMG* electromyographic, *maint.* maintenance treatment, *No.* number.

^a^Only study participants who performed the assessments were counted.

^b^Including other cancer diagnoses and diagnoses that were not clearly specified.

^c^If evaluated in a childhood cancer population.

^d^One study did not include this information, but inclusion criteria were at least one year post-surgery and completed adjuvant treatment program (without radiotherapy).

^e^One study did not include this information, but stated 1–24 months after surgery.

^f^(age at surgery: 5–19 years and time since surgery 13–54 months).

*Cardiorespiratory fitness* (also referred to as endurance, aerobic fitness, or aerobic capacity) was evaluated by a total of 68 studies including *n* = 7936 patients/survivors using 7 lab- and field-based assessment methods (Table 1 and Appendix 3). While the most frequently used assessments (maximal cardiopulmonary exercise test (CPET) and 6MWT) were administered in all diagnostic subgroups during all phases of treatment and a very wide age range, no other assessment was applied during treatment. In terms of measurement properties, the 9MWT has shown to be both reliable and valid in the pediatric oncology population.^92,94^

*Muscle strength* (i.e. muscular endurance or power) was evaluated in 57 studies including *n* = 5679 childhood cancer patients and survivors using 16 different laboratory and field-based assessment methods (Table 2 and Appendix 4). Muscle strength was assessed either by laboratory or field tests focusing on the upper and lower extremities as well as several assessments of core and back muscle strength. While leukemia and/or lymphoma patients and off treatment phase were included in all assessments, some researchers included other cancer diagnoses or phases of medical treatment. In addition, a wide range of age groups was assessed. Isokinetic dynamometry, hand-held dynamometry, and repetition maximum tests are the only assessments that have been shown to be reliable with pediatric cancer cohorts.^92,96^

*Speed* (ability to perform a movement within a short period of time^14^) was assessed in five studies using two different assessments, which comprised a total of *n* = 251 childhood cancer survivors aged between 6 and 30 years (Table 3 and Appendix 5). All testing took place after cessation of treatment. Only field tests, namely shuttle run tests, as the 10 × 5m shuttle run and the 4 × 10 m shuttle run and short distance runs, namely a 60 m run test, were administered. Shuttle run tests were not performed with patients who either present with CNS cancer or bone tumors.

*Balance* was assessed in 15 studies using six different tests including a total of *n* = 2412 patients/survivors (Table 4 and Appendix 6). The nature of assessments was based on posturography and non-posturography methods. While posturography was only performed after medical treatment, two studies conducted balance tests during treatment.^114,148^ Bone tumor patients were only included in one study,^103^ while CNS cancer cohorts were the population of main interest. In terms of age, a wide spectrum including very young children, as well as older adult survivors of childhood cancer (up to an age of 63 years) were analyzed. No information was available on the validity or reliability of any balance assessment in the pediatric oncology population.

*Flexibility* was assessed in 40 studies, applying three different test methods which included a total of *n* = 4309 patients/survivors (Table 5 and Appendix 7). Goniometry, measuring ankle joint range of motion, was performed in most studies including a large number of participants of all ages, with a wide range of diagnoses during all phases of medical treatment. Reliability was analyzed in children with acute lymphoblastic leukemia (ALL) in two studies.^92,158^ In addition, two other flexibility tests measuring hip flexion and trunk flexibility were performed with leukemia/lymphoma, CNS tumor, and other childhood cancer patients and survivors. However, trunk flexibility assessment was only conducted in one study including (young) adults after childhood cancer treatment^62^ while the sit and reach test was applied more often with all age groups during all phases of treatment. Measurement properties were not analyzed within the childhood cancer population.

*Functional mobility* was measured in 29 studies including a total of *n* = 4421 patients using five different assessment methods (Table 6 and Appendix 8). Of these, the Timed Up and Go Test (TUG) was administered in two ways: covering either a 3 m or a 10 m distance. While the TUG 3 m and the Timed Up and Down Stairs Test (TUDS) were applied within several studies, including various childhood cancer diagnoses during all phases of medical treatment, the TUG 10 m was only used with ALL patients during maintenance and/or off treatment.^128,130,131^ Two additional functional tests (stand up from bed rest exam and floor to stand performance test) were both administered within one study each during treatment for childhood cancer.^79,148^ Only the TUG 3 m was performed with older (up to age 64 years) adult survivors of childhood cancer, while all other assessments were conducted with children, adolescents, and young adults. The TUG 3 m demonstrated high validity and reliability^96^ while the TUDS and TUG 10 m both have shown to be reliable.^131^

*Gait analyses* was carried out in 19 studies, using eight different methods including a total of *n* = 545 patients/survivors (Table 7 and Appendix 9). A wide variety of systems were used to assess gait in childhood cancer populations. While few studies used video-recording, partly in combination with force platforms and sometimes electromyography (EMG) measurements, single studies used specific systems, visual observation, or a timed walking test. Except for the EMG analysis of gait and visual observation, all systems assessed gait within various groups of childhood cancer diagnoses. However, only two methods (GAITRite and visual observation) were performed during treatment.^50,148,150^ No information is available on validity and/or reliability of any gait analysis system in the pediatric oncology population.

*Motor performance test batteries* were assessed in 49 studies using 16 different motor test batteries and included a total of 1955 participants (Table 8 and Appendix 10). Most tests were applied in leukemia/lymphoma cohorts after medical treatment. Except for the Functional Mobility Assessment (FMA), which was used in survivors up to age 42 years, all motor test batteries are designed for children and adolescents. Considering all three Bruininks–Oseretsky Test (BOT) versions (BOTMP, BOT-2, BOT-2 SF), the BOT and MOON-Test (Motor performance in pediatric oncology) are the only motor performance test batteries evaluated for feasibility in all diagnosis groups and during all phases of cancer treatment and with young adults. The Gross Motor Function Measure (GFMF) and GFMF-ALL Test Battery are the only assessments that have been evaluated in terms of measurement properties in pediatric oncology populations,^160^ while the University of Québec in Chicoutimi-University of Québec in Montréal (UQAC-UQAM) has been validated using the Jackknife method.^85^

**Table 8:** Summary of study methods assessing motor performance in test batteries in pediatric oncology.

Assessment	No. of studies	Total sample size^a^	Type of cancer				Age in years (range)	Phases of treatment			Validity^c^	Reliability^c^
			Leukemia/lymphoma	Bone tumors	CNS tumors	Other^b^		active	Maint.	Off		
BOT-2	10	327	✓	✓	✓	✓	4–22	✓	–	✓	–	–
BOT-2 SF	6	384	✓	–	–	–	4–18	✓	✓	✓	–	–
m-ABC	5	283	✓	–	–	✓	4.0–19.3	✓	–	✓	–	–
BOTMP	5	164	✓	–	–	✓	1.75–25.2	✓	✓	✓	–	–
m-ABC 2	5	124	✓	✓	–	✓	3–18.7	✓	–	✓	–	–
FMA	4	276	-	✓	–	✓	10.4–42.4	✓	–	✓	–	–
MOON-test	4	141	✓	✓	✓	✓	4–23	✓	✓	✓	–	–
DMT 6–18	4	70	✓	✓	✓	✓	6–17	–	✓	✓	–	–
GMFM	4	62	✓	–	–	–	2–14.6	✓	✓	✓	✓	✓
MOT 4–6	3	22	✓	–	–	✓	3.42–5.42	–	✓	✓	–	–
Lincoln–Oseretzky Motor Development Scale	1	45	✓	–	–	✓	5–14	–	–	✓	–	–
FMS	1	26	✓	–	✓	✓	5–8	–	–	✓	–	–
GMFM – ALL	1	20	✓	–	–	–	2.8–15.9	✓	✓	-	✓	✓
UQAC-UQAM Test Battery	1	20	✓	–	–	–	9–11	–	–	✓	✓	–
Physical fitness battery test adapted by alpha-fitness-test-battery	1	18	✓	–	–	–	7.55 ± 2.43	–	–	✓	–	–
FITNESSGRAM	1	10	✓	–	✓	✓	14.0–18.0	✓	–	✓	–	-

*BOT* Bruininks–Oseretsky Test, *SF* short form, *m-ABC* Movement Assessment Battery for Children, *BOTMP* Bruininks–Oseretsky Test of Motor Proficiency, *FMA* functional mobility assessment, *MOON* motor performance in pediatric oncology, *DMT* Deutscher Motorik Test, *GMFM* gross motor function measure, *MOT* Motoriktest für Kinder, *FMS* fundamental movement skills test battery, *ALL* acute lymphoblastic leukemia, *UQAC-UQAM* University of Québec in Chicoutimi-University of Québec in Montréal, *maint.* maintenance treatment, *No.* number.

^a^Only study participants who performed the assessments were counted.

^b^Including other cancer diagnoses and diagnoses that were not clearly specified.

^c^If evaluated in a childhood cancer population.

## Discussion

This systematic review summarizes the available studies assessing physical performance and function in pediatric cancer patients and survivors. Based on the included 149 studies with 11,639 participants and 63 different assessment tools, we found important characteristics of the distribution and characteristics of the assessments (Table 9). The majority of studies (45.6%) assessing physical or functional performance evaluated cardiorespiratory fitness as an outcome. The 68 studies testing for cardiorespiratory fitness using seven different assessment tools highlight a high homogeneity in the choice of methods. Flexibility was also frequently examined with very uniform assessments. In contrast, muscle strength tests and motor performance batteries have also been evaluated in a high number of studies (57 resp. 49), although with enormous variation in assessment tools. Therefore, the idea of harmonizing physical and functional performance assessments arises to improve comparability of study results. However, harmonization does not seem appropriate nor reasonable across all pediatric cancer types, age groups, treatment phases, and research questions.

**Table 9:** Summary of the main findings.

Main findings are…
1. Physical function and performance were mostly evaluated after medical treatment.
2. Leukemia patients formed the most examined group while solid tumors were less studied.
3. Cardiorespiratory fitness and muscle strength were the physical outcomes of main interest.
4. Assessments with the highest number of participants were
• 6 MWT (*n* = 6180 in 26 studies)
• Grip strength (*n* = 4451 in 27 studies)
• TUG 3 m (*n* = 4283 in 25 studies).
5. Most assessments have not been evaluated for validity and reliability in pediatric cancer populations.

*6MWT* 6-minute walk test, *TUG* timed up and go test.

Speed as a physical performance measure has rarely been evaluated. It can be hypothesized that speed, assessed via shuttle run or other running tests, is difficult to assess during cancer treatment, because children are in a reduced overall condition during cancer treatment. In addition, the health benefits of speed for children and young people appear to be less prominent in the literature than cardiorespiratory fitness and muscle strength^167^ and are therefore less focused in children with and after cancer.

In terms of treatment phase, most studies (66%) have been conducted after cessation of cancer therapy with childhood cancer survivors. The evaluation of persistent physical limitations is of great importance, as they may be limiting to working ability and participation.^168^ Nevertheless, a continuous monitoring of physical performance should be carried out from the time of diagnosis in order to detect physical limitations at an early stage and prevent further deterioration in a sense of early rehabilitation. At the same time, assessment of physical performance from diagnosis onward is important to determine the need for structured exercise. However, since physical fitness, medical side effects, and motivation vary considerably over the course of the therapy, and are dependent on age, diagnoses, and cancer stage, assessment tools evaluating physical performance and function in children with cancer have to fulfill many requirements. To be feasible and safe, different assessments might be chosen according to different groups of patients.

In terms of sample size, eight tests should be highlighted as they were performed by more than 1000 children each, namely grip and isokinetic dynamometry (muscle strength), 6MWT and maximum CPET (cardiorespiratory fitness), goniometry and sit and reach (flexibility), TUG 3 (functional mobility), and SOT (balance). Of those, the 6MWT (*n* = 6180), grip strength (*n* = 4451), and TUG 3 m (*n* = 4283) were the tests with the greatest number of participants. This fact suggests that those outcomes are of specific interest in pediatric oncology as scientists and clinicians seem specifically concerned about their patients’ ability to perform everyday activities, since functional mobility as well as walking capacity measured with the 6MWT are considered important prerequisites to perform physically activities of everyday life.^46,169,170^

Concerning the motor test batteries, geographical differences are noticeable. It can be assumed that countries use tests for which reference values of healthy kindergarten and schoolchildren are available. Especially for younger children motor performance test batteries seem to be appropriate to generate an overview of age-related motor development in comparison with age-related reference values. However, generating a database with reference values for children during and after cancer treatment could be helpful to evaluate skills in the context of cancer treatment.

Measurement properties such as validity or reliability of assessments are rarely available for the population of pediatric cancer patients or survivors. With reference to the period covered by this search, only the 9MWT, TUG 3 m, GMFM, and GMFM-ALL have been tested for validity and reliability, while others (hand held dynamometry, isokinetic dynamometry, repetition maximum test, shuttle run, gonimetry, TUDS, TUG 10 m, and UQAC-UQAM test battery) have been tested for reliability within this population. In addition, it might be useful to examine the quality criteria for children with chronic conditions in general since various chronic health conditions are present during/after pediatric cancer treatment ranging from endocrinological to orthopedic and psychosocial problems.^171^ Moreover, information on quality criteria assessed with healthy children may also be helpful while choosing appropriate assessments. Cooperation of interdisciplinary professional societies and scientists could contribute to a joint evaluation of the quality criteria for chronically ill and disabled children.

Overall, due to the variety of assessments used and the small cohorts often found in childhood cancer studies, the significance of studies on physical and functional performance is very limited. Furthermore, it is hypothesized that children with severe physical limitations have been excluded from many tests that have originally been developed for healthy children.

### Limitations and strength

The strength of this article lies in the comprehensive research, systematic elaboration, and overview of all methods used to test physical performance in the context of pediatric oncology. The inclusion of interventional as well as observational studies allows a complete listing and a clear focus on the assessment tools. Although the lack of a rating and recommendation of the assessments seems to be a weakness, this evaluation was deliberately avoided. Recommendations based on single studies with large sample sizes, personal experiences, or geographical preferences appear inappropriate, as the objective presentation of the assessments were the primary aim. Instead, the results enable researchers and practitioners to select methods from this paper that correspond to their individual research questions or everyday practice and inform researchers about urgent research questions. The elaboration of recommendations and contraindications for the individual therapy phases, cancer types, and study settings as well as the evaluation of the quality criteria in the target group of children with cancer should be the subject of further research in the context of an international consensus. Methodological limitations include that no distinction has been made between diagnosis subgroups (i.e. myeloid and lymphoblastic leukemia) due to lack of detailed descriptions within studies included. The reader should be aware that the frequency with which an assessment is used does not allow any direct conclusions to be drawn about the quality and suitability of the assessment. Rather, the publication date of the assessment, national availability, and translations, as well as material and costs may also have an influence.

### Clinical implication

This systematic review identified 149 studies assessing any category of physical or functional performance in childhood cancer patients or survivors. However, the evidence for the effectiveness of interventions to improve aspects of physical performance is very limited.^9,172^ This might be related to the methodological quality of intervention studies. However, using standardized tools to assess physical and functional performance in defined subgroups of pediatric cancer patients and survivors (i.e. children with ALL during treatment) would enable meta-analyses of single cohorts and overall improve the significance of studies. Apart from clinical research, clinicians, exercise physiologists, and physiotherapists may choose assessment tools presented here with regard to their individual needs and objectives.

### Future research

Future research should focus on evaluating the measurement properties of methods in pediatric cancer populations and children with other chronic diseases. In addition, building an international recommendation statement for assessments in smaller subgroups of pediatric cancer patients and survivors could be a valuable contribution to the current knowledge. Another important step is to generate a database with standard values of children and adolescents suffering from cancer. This could help to compare measures from research and clinical work with children with other chronic conditions, identify impairments and react with early interventions to improve cancer treatment and decrease negative side effects. To expand existing knowledge about leukemia patients to other diagnoses, cancer types like neuroblastoma, retinoblastoma, renal tumors, or soft tissue sarcoma should be tested for physical performance limitations to evaluate their special needs. Furthermore, acute and maintenance treatment phases are less studied but might be of special interest to prevent physical performance deconditioning. And finally, since survivors of childhood cancer can experience very heterogeneous late sequelae, a transferability of the test applications to children with heart or lung diseases, metabolic diseases, or other chronic conditions is conceivable and should be verified in future research projects.

## Supplementary information

Appendixes All
