# Supplementary material for: Physical and functional performance assessment in pediatric oncology: a systematic review
Source: Pediatr Res. 2021 Apr 15;91(4):743–56. doi: 10.1038/s41390-021-01523-5 (PMC9064803; doi:10.1038/s41390-021-01523-5)
Supplement: Supplementary file 1 — Appendixes All [file 41390_2021_1523_MOESM1_ESM.pdf]

**Appendix 1.** Search terms

**Pubmed (Title, Abstract)**

(child [MeSH] OR child* [TIAB] OR adolescent [MeSH] OR AYA [TIAB] OR pediatr* [TIAB] OR paediatr* [TIAB] OR pediatrics [MeSH] OR juvenil* [TIAB] OR infancy [TIAB]) AND (neoplasms [MeSH] OR cancer* [TIAB] OR oncolog* [TIAB] OR tumor* [TIAB] OR tumour* [TIAB] OR leukemia* [TIAB] OR leukemia [MeSH] OR lymphoma [MeSH] OR Hodgkin [TIAB] OR sarcoma [MeSH] OR osteosarcoma [TIAB] OR wilms tumor [MeSH] OR neuroblastoma [TIAB] OR rhabdomyosarcoma [TIAB] OR fibrosarcoma [TIAB] OR hepatoblastoma [TIAB] OR PNET [TIAB] OR medulloblastoma [TIAB] OR retinoblastoma [TIAB] OR glioma [TIAB] OR teratom* [TIAB] OR myeloproliferative disease [TIAB] OR myelodysplastic syndrome [TIAB] OR Ependymoma [TIAB] OR Carcinoma [TIAB] OR Germinom* [TIAB] OR Dysgerminom* [TIAB] OR bone marrow transplant* [TIAB] OR stem cell transplant* [TIAB]) AND (cardiorespiratory fitness [MeSH] OR cardiorespiratory [TIAB] OR cardiopulmonary [TIAB] OR physical endurance [MeSH] OR physical fitness [MeSH] OR exercise test [MeSH] OR exercise tolerance [MeSH] OR spiroergometry [TIAB] OR walk test [MeSH] OR fitness [TIAB] OR endurance [TIAB] OR physical performance [TIAB] OR aerobic performance [TIAB] OR anaerobic performance [TIAB] OR VO2max [TIAB] OR physical capacity [TIAB] OR exercise capacity [TIAB] OR aerobic capacity [TIAB] OR anaerobic capacity [TIAB] OR cycle ergomet* [TIAB] OR treadmill test [TIAB] OR muscle strength [MeSH] muscular strength [TIAB] OR muscle power [TIAB] OR muscular power [TIAB] OR Hand strength [MeSH] OR strength dynamometer [TIAB] OR upper body strength [TIAB] OR leg strength [TIAB] OR sit to stand [TIAB] OR one repetition maximum [TIAB] OR flexibility [TIAB] OR range of motion, articular [MeSH] OR ROM [TIAB] OR flexion [TIAB] OR motor skills [MeSH] OR motor performance [TIAB] OR motor function [TIAB] OR motor coordination [TIAB] OR psychomotor performance [MeSH] OR motor skill* [TIAB] OR coordination test [TIAB] OR Walking speed [MeSH] OR running speed [TIAB] OR Running performance [TIAB] OR reaction time [MeSH] OR agility [TIAB] OR walking velocity [TIAB] OR running velocity [TIAB] OR functional mobility [TIAB] OR motor competence [TIAB] OR motor ability [TIAB] OR functional movement [TIAB] OR gait [MeSH] OR gait pattern* [TIAB] OR gait analysis [TIAB] OR postural balance [MeSH] OR walking efficiency [TIAB] OR postural sway [TIAB] OR stride variability [TIAB] OR step variability [TIAB] OR kinematics [TIAB])

**Cochrane (Title, Abstract, Keywords)**

(mh child OR child* OR mh adolescent OR AYA OR pediatr* OR paediatr* OR mh pediatrics OR juvenil* OR infancy ) AND (mh neoplasms OR cancer* OR oncolog* OR tumor* OR tumour* OR leukemia* OR mh leukemia OR mh lymphoma OR Hodgkin OR mh sarcoma OR osteosarcoma OR mh wilms tumor OR neuroblastoma OR rhabdomyosarcoma OR fibrosarcoma OR hepatoblastoma OR PNET OR medulloblastoma OR retinoblastoma OR glioma OR teratom* OR myeloproliferative disease OR myelodysplastic syndrome OR Ependymoma OR Carcinoma OR Germinom* OR Dysgerminom* OR bone marrow transplant* OR stem cell transplant*) AND (mh cardiorespiratory fitness OR cardiorespiratory OR cardiopulmonary OR mh physical endurance OR mh physical fitness OR mh exercise test OR mh exercise tolerance OR spiroergometry OR mh walk test OR fitness OR endurance OR physical performance OR aerobic performance OR anaerobic performance OR VO2max OR physical capacity OR exercise capacity OR aerobic capacity OR anaerobic capacity OR cycle ergomet* OR treadmill test OR mh muscle strength muscular strength OR muscle power OR muscular power OR mh Hand strength OR strength dynamometer OR upper body strength OR leg strength OR sit to stand OR one repetition maximum OR flexibility OR mh range of motion, articular OR ROM OR flexion OR mh motor skills OR motor performance OR motor function OR motor

coordination OR mh psychomotor performance OR motor skill* OR coordination test OR mh Walking speed OR running speed OR Running performance OR mh reaction time OR agility OR walking velocity OR running velocity OR functional mobility OR motor competence OR motor ability OR functional movement OR mh gait OR gait pattern* OR gait analysis OR mh postural balance OR walking efficiency OR postural sway OR stride variability OR step variability OR kinematics)

**SportDiscus (Title, Abstract)**

AB ( (child* OR adolescen* OR AYA OR pediatr* OR paediatr* OR pediatrics OR juvenil* OR infancy ) AND AB ( neoplasm* OR cancer* OR oncolog* OR tumor* OR tumour* OR leukemia* OR OR lymphoma OR Hodgkin OR sarcoma OR osteosarcoma OR wilms tumor OR neuroblastoma OR rhabdomyosarcoma OR fibrosarcoma OR hepatoblastoma OR PNET OR medulloblastoma OR retinoblastoma OR glioma OR teratom* OR myeloproliferative disease OR myelodysplastic syndrome OR Ependymoma OR Carcinoma OR Germinom* OR Dysgerminom* OR bone marrow transplant* OR stem cell transplant* ) AND AB ( cardiorespiratory OR cardiopulmonary OR physical endurance OR physical OR exercise test* OR exercise tolerance OR spiroergometr* OR walk* test* OR fitness OR endurance OR physical performance OR aerobic performance OR anaerobic performance OR VO2max OR physical capacity OR exercise capacity OR aerobic capacity OR anaerobic capacity OR cycle ergomet* OR treadmill test* OR muscle strength OR muscular strength OR muscle power OR muscular power OR Hand strength OR strength dynamometer OR upper body strength OR leg strength OR sit to stand OR one repetition maximum OR flexibility OR range of motion OR ROM OR flexion OR motor skills OR motor performance OR motor function OR motor coordination OR psychomotor performance OR motor skill* OR coordination test* OR Walking speed OR running speed OR Running performance OR reaction time OR agility OR walking velocity OR running velocity OR functional mobility OR motor competence OR motor ability OR functional movement OR gait OR postural balance OR walking efficiency OR postural sway OR stride variability OR step variability OR kinematics) ) OR TI ( (child* OR adolescen* OR AYA OR pediatr* OR paediatr* OR pediatrics OR juvenil* OR infancy ) AND TI ( neoplasm* OR cancer* OR oncolog* OR tumor* OR tumour* OR leukemia* OR OR lymphoma OR Hodgkin OR sarcoma OR osteosarcoma OR wilms tumor OR neuroblastoma OR rhabdomyosarcoma OR fibrosarcoma OR hepatoblastoma OR PNET OR medulloblastoma OR retinoblastoma OR glioma OR teratom* OR myeloproliferative disease OR myelodysplastic syndrome OR Ependymoma OR Carcinoma OR Germinom* OR Dysgerminom* OR bone marrow transplant* OR stem cell transplant* ) AND TI ( cardiorespiratory OR cardiopulmonary OR physical endurance OR physical OR exercise test* OR exercise tolerance OR spiroergometr* OR walk* test* OR fitness OR endurance OR physical performance OR aerobic performance OR anaerobic performance OR VO2max OR physical capacity OR exercise capacity OR aerobic capacity OR anaerobic capacity OR cycle ergomet* OR treadmill test* OR muscle strength OR muscular strength OR muscle power OR muscular power OR Hand strength OR strength dynamometer OR upper body strength OR leg strength OR sit to stand OR one repetition maximum OR flexibility OR range of motion OR ROM OR flexion OR motor skills OR motor performance OR motor function OR motor coordination OR psychomotor performance OR motor skill* OR coordination test* OR Walking speed OR running speed OR Running performance OR reaction time OR agility OR walking velocity OR running velocity OR functional mobility OR motor competence OR motor ability OR functional movement OR gait OR postural balance OR walking efficiency OR postural sway OR stride variability OR step variability OR kinematics) )

**Appendix 2:** Description of studies included in the systematic review.

| Author | Publication year | Sample size | Assessments | Type of cancer | Phase of treatment |
|---|---|---|---|---|---|
| **Akyay et al.** | 2014 | 33 | Muscular strength: Grip strength<br>Functional mobility: TUG 3m | Leukemia | Mixed |
| **Bastian et al.** | 1998 | 5 | Gait: Video recording | CNS | No information |
| **Bell et al.** | 2006 | 35 | Cardiorespiratory fitness: Maximal CPET | Leukemia | Off treatment |
| **Benedetti et al.** | 2016 | 16 | Gait: Video-recording and force platforms | Bone tumor | Off treatment |
| **Beulertz et al.** | 2013 | 24 | Motor performance test battery: MOT, DMT | Mixed | Mixed |
| **Beulertz et al.** | 2016a | 13 | Cardiorespiratory fitness: 6MWT<br>Flexibility: Goniometry<br>Gait: Microgate Optogait<br>Motor performance test battery: DMT | Mixed | Off treatment |
| **Beulertz et al.** | 2016b | 33 | Motor performance test battery: MOT, DMT | Mixed | Mixed |
| **Bianco et al.** | 2014 | 18 | Muscular strength: Sit-up test, Standing broad Jump, Grip strength<br>Speed: Shuttle Run<br>Motor performance test battery: Physical fitness battery | Hematologic malignancies | Off treatment |
| **Black et al.** | 1998 | 56 | Cardiorespiratory fitness: Maximal CPET | Leukemia | Off treatment |
| **Braam et al.** | 2016 | 60 | Cardiorespiratory fitness: Maximal CPET<br>Muscular strength: Hand Held Dynamometry | Mixed | Mixed |
| **Braam et al.** | 2018 | 68 | Cardiorespiratory fitness: Maximal CPET<br>Muscular strength: Hand Held Dynamometry, Grip strength | Mixed | Mixed |
| **Braam et al.** | 2015 | 61 | Cardiorespiratory fitness: Maximal CPET | Mixed | Mixed |
| **Brinkman et al.** | 2018 | 306 | Cardiorespiratory fitness: 6MWT<br>Muscular strength: Isokinetic Dynamometry, Grip strength<br>Balance: SOT<br>Flexibility: Sit and reach<br>Functional mobility: TUG 3m | CNS | Off treatment |
| **Carty et al.** | 2009a | 20 | Gait: Video-recording and force platforms | Bone tumor | No information |
| **Carty et al.** | 2009b | 20 | Muscular strength: Manual Muscle Test<br>Flexibility: Goniometry | Bone tumor | Off treatment |
| **Corr et al.** | 2017 | 14 | Muscular strength: Hand Held Dynamometry<br>Flexibility: Goniometry<br>Motor performance test battery: FMA | Lower extremity sarcoma | Active treatment |
| **Cortés-Reyes et al.** | 2013 | 7 | Cardiorespiratory fitness: 6MWT<br>Muscular strength: Sit up test<br>Flexibility: Sit and reach<br>Motor performance test battery: GMFM | Leukemia | Mixed |
| **Cox et al.** | 2018 | 107 | Cardiorespiratory fitness: 6MWT<br>Muscular strength: Hand Held Dynamometry, Grip strength<br>Flexibility: Goniometry<br>Motor performance test battery: BOT 2SF | Leukemia | Active treatment |
| **Däggelmann et al.** | 2017 | 22 | Motor performance test battery: MOT, DMT | Mixed | Off treatment |
| **Davis et al.** | 2010 | 15 | Motor performance test battery: BOT 2 | CNS | Active treatment |
| **De Caro et al.** | 2006 | 84 | Cardiorespiratory fitness: Maximal CPET | Mixed | Off treatment |
| **DeFeo et al.** | 2020 | 1695 | Cardiorespiratory fitness: 6MWT<br>Muscular strength: Isokinetic dynamometry, Grip strength<br>Flexibility: Sit and reach, Goniometry<br>Functional mobility: TUG 3m | Mixed | Off treatment |
| **Deisenroth et al.** | 2016 | 40 | Muscular strength: Hand Held Dynamometry | Mixed | Active treatment |
| **Dubnov-Raz et al.** | 2015 | 20 | Cardiorespiratory fitness: Maximal CPET | Mixed | Off treatment |

| | | | | | |
|---|---|---|---|---|---|
| **Ehrhardt et al.** | 2017 | 200 | Cardiorespiratory fitness: 6MWT<br>Muscular strength: Isokinetic dynamometry, Grip strength<br>Balance: SOT<br>Flexibility: Goniometry, Sit and reach<br>Functional mobility: TUG 3m | Lymphoma | Off treatment |
| **Esbenshade et al.** | 2014 | 17 | Cardiorespiratory fitness: 6MWT<br>Muscular strength: Hand Held Dynamometry, Grip strength<br>Flexibility: Goniometry, Sit and reach<br>Motor performance test battery: BOT 2SF | Leukemia | Maintenance |
| **Fiorillo et al.** | 2010 | 16 | Gait: Video recording | CNS | Off treatment |
| **Fiuza-Luces et al.** | 2017 | 49 | Cardiorespiratory fitness: Maximal CPET<br>Muscular strength: Repetition Maximum<br>Functional mobility: TUG 3m, TUDS | Mixed | Mixed |
| **Fuchs et al.** | 2003 | 6 | Gait: Video-recording and force platforms | Bone tumor | Off treatment |
| **Galea et al.** | 2004 | 79 | Balance: Balance test on force platform | Leukemia | Off treatment |
| **Gerber et al.** | 2006 | 32 | Cardiorespiratory fitness: 6MWT<br>Muscular strength: Manual Muscle Test, Grip strength<br>Flexibility: Goniometry<br>Motor performance test battery: BOT 2 | Mixed | Off treatment |
| **Gilchrist et al.** | 2016 | 52 | Cardiorespiratory fitness: 6MWT<br>Flexibility: Goniometry<br>Gait: GAITRite | Mixed | Active treatment |
| **Gilchrist & Tanner** | 2018 | 65 | Motor performance test battery: BOT 2 | Mixed | Active treatment |
| **Gilliam et al.** | 2011 | 12 | Cardiorespiratory fitness: PACER<br>Muscular strength: Hand Held Dynamometry, Chair-Stand Test, Lateral Step Test | Mixed | Off treatment |
| **Ginsberg et al.** | 2007 | 91 | Motor performance test battery: FMA | Bone tumor | Off treatment |
| **Gohar et al.** | 2011 | 9 | Motor performance test battery: GMFM | Leukemia | Active treatment |
| **Götte & Kesting et al.** | 2013 | 33 | Motor performance test battery: MOON | Mixed | mixed |
| **Götte et al.** | 2015 | 47 | Motor performance test battery: MOON | Mixed | Active treatment |
| **Götte & Kesting et al.** | 2018 | 40 | Motor performance test battery: MOON | Mixed | Active treatment |
| **Hamari et al.** | 2019 | 36 | Motor performance test battery: mABC 2 | Mixed | Active treatment |
| **Harten et al.** | 1984 | 45 | Motor performance test battery: Lincoln-Oseretzky Motor Development Scale | Mixed | Off treatment |
| **Hartman et al.** | 2009 | 51 | Flexibility: Goniometry<br>Motor performance test battery: mABC | Leukemia | Active treatment |
| **Hartman et al.** | 2010 | 34 | Motor performance test battery: mABC | Leukemia | Off treatment |
| **Hartman et al.** | 2013 | 34 | Cardiorespiratory fitness: 6MWT<br>Flexibility: Goniometry<br>Motor performance test battery: mABC 2 | Leukemia | Off treatment |
| **Hartman et al.** | 2018 | 71 | Cardiorespiratory fitness: 6MWT<br>Muscular strength: Sit up test, Back estension test, Push-up test, Vertical jump, Grip strength<br>Flexibility: Sit and reach, Side-bending | Mixed | Off treatment |
| **Hartman et al.** | 2006 | 128 | Motor performance test battery: mABC | Mixed | Off treatment |
| **Hartman et al.** | 2008 | 92 | Muscular strength: Hand held dynamometry<br>Flexibility: Goniometry | Mixed | Off treatment |
| **Hauser et al.** | 2001 | 38 | Cardiorespiratory fitness: Maximal CPET | Leukemia | Off treatment |
| **Henderson et al.** | 2012 | 38 | Flexibility: Goniometry | Bone tumor | Off treatment |
| **Hillmann et al.** | 2001 | 7 | Gait: Video-recording, force plates and EMG | Mixed | Off treatment |
| **Hillmann et al.** | 2000 | 43 | Gait: Video-recording, force plates and EMG | Mixed | Off treatment |

| | | | | | |
|---|---|---|---|---|---|
| **HOff treatmentman et al.** | 2013 | 183 | Cardiorespiratory fitness: 6MWT<br>Muscular strength: Isokinetic dynamometry, Grip strength<br>Functional mobility: TUG 3m | Mixed | Off treatment |
| **Hogarty et al.** | 2000 | 31 | Cardiorespiratory fitness: Maximal CPET | Mixed | Off treatment |
| **Hovi et al.** | 1993 | 43 | Muscular strength: Isometric dynamometry, Sit up test, Push-up test | Leukemia | Off treatment |
| **Hovi et al.** | 2010 | 83 | Muscular strength: Sit up test, Repeated Squatting, Back extension, Leg lift<br>Speed: Shuttle run<br>Flexibility: Sit and reach | Mixed | Off treatment |
| **Howell et al.** | 2018 | 78 | Muscular strength: Sit up test, Push up test, Grip strength | Mixed | Off treatment |
| **Hung et al.** | 2017 | 13 | Cardiorespiratory fitness: 6MWT<br>Motor performance test battery: BOT 2SF | Leukemia | Off treatment |
| **Järvelä et al.** | 2010 | 21 | Cardiorespiratory fitness: Maximal CPET<br>Muscular strength: Sit up test, Repeated Squatting, Back extension, Vertical jump, Shoulder lift, Grip strength | Leukemia | Off treatment |
| **Jenney et al.** | 1995 | 70 | Cardiorespiratory fitness: Maximal CPET | Leukemia | Off treatment |
| **Johnson et al.** | 1997 | 13 | Cardiorespiratory fitness: Submaximal CPET, Maximal CPET | No information | Off treatment |
| **Joyce et al.** | 2011 | 493 | Muscular strength: Isometric dynamometry, Grip strength | Leukemia | Off treatment |
| **Kabak et al.** | 2016 | 13 | Cardiorespiratory fitness: 6MWT<br>Muscular strength: Chair-stand test, Grip strength<br>Functional mobility: TUG 3m, TUDS, Stand up from bed rest | Mixed | Active treatment |
| **Kandula et al.** | 2018 | 65 | Motor performance test battery: mABC | No information | Off treatment |
| **Keats & Culos-Reed** | 2008 | 10 | Motor performance test battery: FITNESSGRAMM | Mixed | Mixed |
| **Kesting et al.** | 2015 | 21 | Motor performance test battery: MOON | Bone tumor | Off treatment |
| **Konczak et al.** | 2005 | 22 | Balance: SOT | CNS | Off treatment |
| **Lam et al.** | 2018 | 70 | Muscular strength: Grip strength | Mixed | Off treatment |
| **Leone et al.** | 2014 | 20 | Motor performance test battery: UQAC-UQAM | Leukemia | Off treatment |
| **Long et al.** | 2017 | 20 | Cardiorespiratory fitness: Maximal CPET<br>Muscular strength: Repetition maximum, Sit up test, Repeated squatting, Push-up test | Mixed | Off treatment |
| **Long et al.** | 2018 | 13 | Cardiorespiratory fitness: Maximal CPET, Submaximal CPET<br>Muscular strength: Repetition maximum, Sit up test, Repeated squatting, Push-up test | Mixed | Off treatment |
| **Luca De et al.** | 2013 | 37 | Motor performance test battery: BOT 2SF, mABC 2 | Leukemia | Off treatment |
| **Malicka et al.** | 2020 | 34 | Speed: 60m run | Leukemia | Off treatment |
| **Malicka et al.** | 2019 | 71 | Speed: 60m run | Mixed | Off treatment |
| **Manchola-González et al.** | 2019 | 19 | Cardiorespiratory fitness: Maximal CPET<br>Muscular strength: Grip strength<br>Flexibility: Sit and reach<br>Functional mobility: TUG 3m, TUDS | Leukemia | Off treatment |
| **Marchese, Chiarello et al.** | 2004a | 28 | Cardiorespiratory fitness: 9MWT<br>Muscular strength: Hand held dynamometry<br>Flexibility: Goniometry<br>Functional mobility: TUDS | Leukemia | Maintenance |
| **Marchese, Ogle et al.** | 2004b | 18 | Cardiorespiratory fitness: 9MWT<br>Functional mobility: TUG 3m, TUDS | Bone tumor | Off treatment |
| **Marchese et al.** | 2007 | 94 | Motor performance test battery: FMA | Mixed | Off treatment |
| **Marchese et al.** | 2006 | 68 | Cardiorespiratory fitness: 9MWT<br>Flexibility: Goniometry<br>Functional mobility: TUG 3m, TUDS | Lower extremity sarcoma | Off treatment |

| | | | | | |
|---|---|---|---|---|---|
| **Marchese et al.** | 2003 | 8 | Muscular strength: Hand held dynamometry<br>Functional mobility: TUG 3m | Leukemia | Active treatment |
| **Marchese et al.** | 2008 | 33 | Flexibility: Goniometry<br>Functional mobility: TUDS | Leukemia | Mixed |
| **Matthys et al.** | 1993 | 35 | Cardiorespiratory fitness: Maximal CPET | Mixed | Off treatment |
| **McKenzie et al.** | 2000 | 34 | Cardiorespiratory fitness: Maximal CPET, Wingate anaerobic test | Solid tumor | Off treatment |
| **Mitchell et al.** | 2002 | 17 | Motor performance test battery: BOTMP | Neuroblastoma | Maintenance |
| **Varedi et al.** | 2018 | 365 | Cardiorespiratory fitness: 6MWT<br>Functional mobility: TUG 3m | Leukemia | Off treatment |
| **Moyer-Mileur et al.** | 2009 | 14 | Cardiorespiratory fitness: PACER<br>Muscular strength: Push up test<br>Flexibility: Sit and reach<br>Functional mobility: TUG 3m, TUDS | Leukemia | Maintenance |
| **Müller et al.** | 2017 | 84 | Balance: Balance test on force platform<br>Gait: Video recording | Mixed | N/A |
| **Muratt et al.** | 2011 | 10 | Muscular strength: Isokinetic dynamometry | Leukemia | Maintenance |
| **Nama et al.** | 2020 | 72 | Muscular strength: Manual muscle test<br>Functional mobility: TUG 3m<br>Motor performance test battery: BOT 2 | Mixed | Active treatment |
| **Naumann et al.** | 2015 | 26 | Motor performance test battery: FMS | Mixed | Off treatment |
| **Ness et al.** | 2007 | 75 | Cardiorespiratory fitness: 2MWT<br>Muscular strength: Hand held dynamometry, Grip strength<br>Functional mobility: TUG 3m | Leukemia | Off treatment |
| **Ness et al.** | 2015a | 365 | Cardiorespiratory fitness: submaximal CPET<br>Muscular strength: Isokinetic dynamometry, Grip strength<br>Balance: SOT<br>Flexibility: Goniometry, Sit and reach | Leukemia | Off treatment |
| **Ness et al.** | 2012 | 415 | Cardiorespiratory fitness: 6MWT<br>Muscular strength: Isokinetic dynamometry<br>Balance: SOT<br>Flexibility: Goniometry<br>Functional mobility: TUG 3m | Leukemia | Off treatment |
| **Ness et al.** | 2013 | 475 | Cardiorespiratory fitness: 6MWT<br>Balance: SOT<br>Functional mobility: TUG 3m | Extracranial solid tumor | Off treatment |
| **Ness et al.** | 2015b | 109 | Cardiorespiratory fitness: 6MWT<br>Muscular strength: Hand held dynamometry, Grip strength<br>Flexibility: Goniometry<br>Motor performance test battery: BOT 2SF | Leukemia | Active treatment |
| **Ness et al.** | 2010 | 156 | Muscular strength: Hand held dynamometry, Grip strength<br>Balance: Berg balance test | CNS | Off treatment |
| **Ness et al.** | 2014 | 42 | Flexibility: Goniometry<br>Motor performance test battery: FMA | Bone tumor | Off treatment |
| **Nielsen et al.** | 2018 | 75 | Cardiorespiratory fitness: Maximal CPET<br>Muscular strength: Chair-Stand test, Grip strength<br>Balance: Flamingo balance test<br>Functional mobility: TUG 3m | Mixed | Active treatment |
| **Oschwald et al.** | 2019 | 16 | Cardiorespiratory fitness: 2MWT<br>Muscular strength: Hand held dynamometry<br>Flexibility: Goniometry<br>Functional mobility: TUG 3m<br>Gait: 10m Walk test | Mixed | Mixed |
| **Oswald & Bo** | 2020 | 13 | Motor performance test battery: mABC 2 | Leukemia | Off treatment |
| **Ovans et al.** | 2018 | 15 | Cardiorespiratory fitness: 6MWT | CNS | Off treatment |
| **Papalia et al.** | 2020 | 10 | Cardiorespiratory fitness: Submaximal CPET | CNS | Off treatment |

| | | | | | |
|---|---|---|---|---|---|
| **Pesenti et al.** | 2018 | 15 | Gait: Video-recording and force platforms | Bone tumor | Off treatment |
| **Pihkala et al.** | 1995 | 30 | Cardiorespiratory fitness: Maximal CPET | Mixed | Off treatment |
| **Piscione et al.** | 2017 | 28 | Cardiorespiratory fitness: Maximal CPET<br>Motor performance test battery: BOT 2 | CNS | Off treatment |
| **Piscione et al.** | 2014 | 30 | Motor performance test battery: BOT 2 | CNS | Off treatment |
| **Ramchandren et al.** | 2009 | 37 | Motor performance test battery: BOT 2 | Leukemia | Off treatment |
| **Reindres-Messelink et al.** | 1999 | 17 | Motor performance test battery: mABC | Leukemia | Active treatment |
| **Riggs et al.** | 2017 | 28 | Cardiorespiratory fitness: 6MWT | CNS | Off treatment |
| **Rosenhagen et al.** | 2011 | 23 | Muscular strength: Grip strength | Mixed | Active treatment |
| **Sabel et al.** | 2016 | 13 | Motor performance test battery: BOT 2 | CNS | Off treatment |
| **San Juan et al.** | 2008a | 15 | Cardiorespiratory fitness: Maximal CPET<br>Flexibility: Goniometry<br>Functional mobility: TUDS | Leukemia | Maintenance |
| **San Juan et al.** | 2008b | 8 | Cardiorespiratory fitness: Maximal CPET<br>Muscular strength: Repetition maximum<br>Flexibility: Goniometry<br>Functional mobility: TUG 3m, TUG 10m, TUDS | Leukemia | Off treatment |
| **San Juan et al.** | 2007a | 7 | Cardiorespiratory fitness: Maximal CPET<br>Muscular strength: Repetition maximum<br>Flexibility: Goniometry<br>Functional mobility: TUG 3m, TUG 10m, TUDS | Leukemia | Maintenance |
| **San Juan et al.** | 2007b | 7 | Muscular strength: Repetition maximum<br>Functional mobility: TUG 3m, TUG 10m, TUDS | Leukemia | Maintenance |
| **Schoch et al.** | 2006 | 22 | Balance: SOT | CNS | Off treatment |
| **Schoch et al.** | 2010 | 22 | Balance: Ultrasound-based motion analysis | CNS | Off treatment |
| **Schoenmakers et al.** | 2006 | 18 | Muscular strength: Manual muscle test<br>Motor performance test battery: mABC 2 | Mixed | Mixed |
| **Segerer et al.** | 2017 | 74 | Cardiorespiratory fitness: Maximal CPET | Solid tumor | Off treatment |
| **Senn-Malashonak et al.** | 2019 | 60 | Cardiorespiratory fitness: 6MWT, Maximal CPET<br>Muscular strength: Isometric dynamometry, Grip strength | Mixed | Active treatment |
| **Shore & Shepard** | 1999 | 6 | Cardiorespiratory fitness: Maximal CPET | N/A | Mixed |
| **Slater et al.** | 2015 | 119 | Cardiorespiratory fitness: 6MWT<br>Muscular strength: Grip strength<br>Functional mobility: TUG 3m | Mixed | Off treatment |
| **Smith et al.** | 2014 | 1778 | Cardiorespiratory fitness: 6MWT | Mixed | Off treatment |
| **Smith et al.** | 2013 | 5 | Cardiorespiratory fitness: Maximal CPET<br>Muscular strength: Isokinetic dynamometry | Bone tumor | Off treatment |
| **SteenhOff treatment et al.** | 1993 | 8 | Gait: EMG analysis | Bone tumor | Off treatment |
| **Su et al.** | 2018 | 18 | Cardiorespiratory fitness: 6MWT | Mixed | Off treatment |
| **Syczewska et al.** | 2008 | 88 | Balance: Balance test on force platform | CNS | Off treatment |
| **Syczewska et al.** | 2006 | 41 | Balance: Balance test on force platform<br>Gait: Video-recording and force platform | CNS | Off treatment |
| **Syczewska et al.** | 2010 | 105 | Gait: Video-recording | CNS | Off treatment |
| **Takken et al.** | 2009 | 9 | Cardiorespiratory fitness: Maximal CPET<br>Muscular strength: Hand held dynamometry, Grip strength<br>Functional mobility: TUG 3m, TUDS | Leukemia | Off treatment |
| **Talvensaari et al.** | 1995 | 46 | Cardiorespiratory fitness: Maximal CPET<br>Muscular strength: Isokonetic dynamometry | Mixed | Off treatment |

| | | | | | |
|---|---|---|---|---|---|
| **Tanir & Kuguoglu** | 2013 | 40 | Cardiorespiratory fitness: 9MWT<br>Muscular strength: Isometric dynamometry<br>Flexibility: Goniometry<br>Functional mobility: TUG 3m, TUDS | Leukemia | Off treatment |
| **Tanner et al.** | 2017 | 62 | Balance: Single leg stance<br>Flexibility: Goniometry<br>Functional mobility: Floor to stand performance<br>Gait: Visual observation<br>Motor performance test battery: BOTMP, BOT 2 | Leukemia | Active treatment |
| **Tanner & Hooke** | 2019 | 30 | Flexibility: Goniometry<br>Motor performance test battery: BOT 2 | Leukemia | Off treatment |
| **Tanner et al.** | 2015 | 6 | Cardiorespiratory fitness: 6MWT<br>Muscular strength: Manual muscle test, Hand held dynamometry<br>Flexibility: Goniometry<br>Gait: GAITRite | Mixed | Active treatment |
| **Taskinen et al.** | 2013 | 45 | Muscular strength: Sit-up test, Repeated squatting, Back extension test, Leg lift test<br>Speed: Shuttle run<br>Flexibility: Sit and reach | Leukemia | Off treatment |
| **Tay et al.** | 2017 | 101 | Motor performance test battery: BOT 2SF | Leukemia | Off treatment |
| **Turner-Gomes et al.** | 1996 | 11 | Cardiorespiratory fitness: Wingate anaerobic test, Maximal CPET | Leukemia | Off treatment |
| **van Brussel et al.** | 2006 | 13 | Cardiorespiratory fitness: Wingate anaerobic test, Maximal CPET<br>Muscular strength: Hand held dynamometry | Leukemia | Off treatment |
| **Wallek et al.** | 2018 | 49 | Cardiorespiratory fitness: 6MWT | Mixed | Active treatment |
| **Warner et al.** | 1997 | 56 | Cardiorespiratory fitness: Submaximal CPET, Maximal CPET | Mixed | Off treatment |
| **Wiernikowski et al.** | 2005 | 10 | Motor performance test battery: BOTMP, GMFM | Mixed | Maintenance |
| **Wright et al.** | 2003 | 40 | Flexibility: Goniometry | Leukemia | Mixed |
| **Wright et al.** | 2017 | 17 | Muscular strength: Manual muscle test<br>Flexibility: Goniometry<br>Gait: Video-recording, force plates and EMG | Leukemia | Mixed |
| **Wright & Fairfield** | 2007 | 20 | Motor performance test battery: GMFM ALL | Leukemia | Mixed |
| **Wright et al.** | 2005 | 99 | Motor performance test battery: BOTMP | Leukemia | Off treatment |
| **Wright et al.** | 1999 | 54 | Flexibility: Goniometry | Leukemia | Off treatment |
| **Wright et al.** | 1998 | 36 | Muscular strength: Grip strength<br>Flexibility: Goniometry<br>Motor performance test battery: BOTMP, GMFM | Leukemia | Off treatment |
| **Wurz et al.** | 2014 | 8 | Flexibility: Goniometry, Sit and reach<br>Functional mobility: TUG 3m | Mixed | Off treatment |
| **Zaccara et al.** | 2004 | 13 | Gait: Video-recording and force platform | Germ cell tumor | Off treatment |

2MWT: 2-minute walk test, 6MWT: 6-minute walk test, 9MRWT: 9-minute run-walk test, CNS: central nervous system, CPET: cardio pulmonary exercise test, EMG: electromyography, SOT: sensory organization test, BOT: Bruininks-Oseretsky Test, SF: short form; m-ABC: Movement Assessment Battery for Children, BOTMP: Bruininks-Oseretsky Test of Motor Proficiency, FMA: Functional Mobility Assessment, MOON: Motor Performance in Pediatric Oncology, DMT: Deutscher Motorik Test, GMFM: Gross motor function measure, MOT: Motoriktest für Kinder, FMS: fundamental movement skills test battery, UQAC-UQAM: University of Québec in Chicoutimi-University of Québec in Montréal

**Appendix 3:** Description of study methods assessing cardiorespiratory fitness in pediatric cancer patients and survivors

| Assessment | No. of studies/ total sample size[1] | Type of Cancer (No. participants) | Age range/ mean±SD [y] | Phases of treatment (No. studies) | Validity[2] | Reliability[2] | References |
|---|---|---|---|---|---|---|---|
| **Maximal CPET** *Measurement of maximum aerobic capacity* | 33/1170 | Leukemia (621)<br>Lymphoma (130)<br>CNS tumor (84)<br>Neuroblastoma (19)<br>Retinoblastoma (1)<br>Renal tumor (28)<br>Hepatoblastoma (3)<br>Bone tumor (45)<br>Soft tissue sarcoma (26)<br>Germ cell (1)<br>Mixed (207)<br>*[solid tumor (207)]*<br>Other (5) | 3.5-41 | Active treatment (2)<br>Maintenance (2)<br>Off treatment (24)<br>Mixed (5)<br>*[all phases (4), maint./off treatment (1)]* | No information | No information | Bell et al. 2006; Black et al. 1998; Braam et al. 2015; Braam et al. 2016; Braam et al. 2018; Caro et al. 2006; Dubnov-Raz et al. 2015; Fiuza-Luces et al. 2017; Hauser et al. 2001; Hogarty et al. 2000; Järvelä et al. 2010; Jenney et al. 1995; Johnson et al. 1997; Long et al. 2017; Long et al. 2018; Manchola-González et al. 2019; Matthys et al. 1993; McKenzie et al. 2000; Nielsen et al. 2018; Pihkala et al. 1995; Piscione et al. 2017; San Juan et al. 2007; San Juan, Chamorro-Viña, Maté-Muñoz et al. 2008; San Juan, Chamorro-Viña, Moral et al. 2008; Segerer et al. 2017; Senn-Malashonak et al. 2019; Shore & Shepard 1999; Smith et al. 2013; Takken et al. 2009; Talvensaari et al. 1995; Turner-Gomes et al. 1996; van Brussel et al. 2006; Warner et al. 1997) |
| **6-Minute Walk Test** *Measurement of functional submaximal aerobic capacity via timed walk test (subject walks as fast as possible on a defined straight and flat corridor for 6 min)* | 26/6180 | Leukemia (3231)<br>Lymphoma (1139)<br>CNS tumor (525)<br>Neuroblastoma (33)<br>Renal tumor (30)<br>Bone tumor (152)<br>Soft tissue sarcoma (10)<br>Germ cell tumor (1)<br>Mixed (1054)<br>*[saroma (2), mixed solid (427), non-CNS solid tumor (11), extracranial solid tumor (475), ALL/Wilms tumor/other (6), leukemia/lymphoma (59), bone/soft tissue sarcoma (13), neuroblastoma/Wilms tumor (49), other (12)]* | 3.5-63.8 | Active treatment (7)<br>Maintenance (1)<br>Off treatment (17)<br>Mixed (1) | No information | No information | Beulertz et al. 2016; Brinkman et al. 2018; Cortés-Reyes et al. 2013; Cox et al. 2018; DeFeo et al. 2020; Ehrhardt et al. 2017; Esbenshade et al. 2014; Gerber et al. 2006; Gilchrist & Tanner 2016; Hartman et al. 2013; Hartman et al. 2018; Hoffman et al. 2013; Hung et al. 2017; Ness et al. 2012; Ness et al. 2013; Ness, Kaste et al. 2015; Ovans et al. 2018; Riggs et al. 2017; Senn-Malashonak et al. 2019; Slater et al. 2015; Smith et al. 2014; Su et al. 2018; Tanner et al. 2015; Varedi et al. 2017; Wallek et al. 2018; Yildiz Kabak et al. 2016) |

| | | | | | | | |
|---|---|---|---|---|---|---|---|
| | | Other (5)<br>*[Nasopharynx-CA (1), neuroectodermal tumor (3), N/A (1)]* | | | | | |
| **Submaximal CPET**<br>*Measurement of submaximal aerobic capacity* | 5/457 | Leukemia (409)<br>Lymphoma (4)<br>CNS tumor (19)<br>Neuroblastoma (2)<br>Renal tumor (6)<br>Soft tissue sarcoma (1)<br>Mixed (15)<br>Other (1)<br>*[Undifferentiated rhabdomyosarcoma of the right petrous temporal bone]* | 7-44.6 | Off treatment (5) | No information | No information | Johnson et al. 1997; Long et al. 2018; Ness, DeLany et al. 2015; Papalia et al. 2020; Warner et al. 1997 |
| **9MWT/9MRWT**<br>*Measurement of aerobic capacity via timed walk test, subject runs or walks as fast as possible on a defined straight and flat corridor for 9 min* | 4/154 | Leukemia (68)<br>Bone tumor (18)<br>Mixed (68)<br>*[lower-extremity sarcoma (68)]* | 4-27 | Maintenance (1)<br>Off treatment (3) | Concurrent and construct validity: Spearman rho = 0.63 and 0.50 between 9 min run-walk distance and MSTS and TESS (lower extremity version) scores, resp. in 94 CCS with lower extremity sarcoma aged 10-42years; Longitudinal data at 6, 12 and 18 | Intra- and inter-rater reliability: $r \geq 0.85$ (CCP during maintenance therapy for ALL) (Marchese, Chiarello, & Lange, 2004)<br>Intra- and inter-rater reliability: ICC ≥ 0.97 (23 CCS with lower extremity sarcoma 10-42y) (Marchese et al., 2007) | Marchese et al. 2006; Marchese, Chiarello, & Lange 2004; Marchese, Ogle et al. 2004; Tanir & Kuguoglu 2013 |

| | | | | | | | |
|---|---|---|---|---|---|---|---|
| | | | | | months after limb sparing surgery indicated an increase in distance walked over time ($p$ = 0.04) in 6 CCS with lower extremity sarcoma aged 11-22years (Marchese et al., 2007) | | |
| **Wingate anaerobic test** (and similar 30s-all-out protocols) *Measurement of anaerobic capacity via cycle ergometer, maximum effort for 30s following a short warm-up period* | 3/58 | Leukemia (24)<br>Mixed (34)<br>*[Solid tumor (34)]* | 7.7-23.8 | Off treatment (3) | No information | No information | McKenzie et al. 2000; Turner-Gomes et al. 1996; van Brussel et al. 2006 |
| **2MWT** *Measurement of submaximal aerobic capacity via timed walk test, subject walks as fast as possible on a defined straight* | 2/91 | Leukemia (79)<br>Lymphoma (3)<br>CNS tumor (1)<br>Neuroblastoma (2)<br>Soft tissue sarcoma (2)<br>Germ cell tumor (1)<br>Other (3)<br>[juvenile granulosa cell tumor) (1), malignant peripheral nerve sheath tumor (1), | 6-45 | Off treatment (1)<br>Mixed (1)<br>*[maint./off treatment (1)]* | No information | No information | Ness et al. 2007; Oschwald et al. 2019 |

| | | | | | | | |
|---|---|---|---|---|---|---|---|
| *and flat corridor for 2 min* | | undifferentiated sarcoma of the liver (1)] | | | | | |
| **PACER Shuttle Run Test** *Measurement of maximum aerobic capacity, subject runs back and forth across a 20m space at a specified pace that gets faster each minute as long as possible* | 2/26 | Leukemia (21)<br>CNS tumor (5) | 4-18 | Maintenance (1)<br>Off treatment (1) | No information | No information | Gilliam et al. 2011; Moyer-Mileur et al. 2009 |

ALL: acute lymphoblastic leukemia, CCP: childhood cancer patients, CCS: childhood cancer survivors, MSTS: Musculoskeletal Tumor Society Rating Scale, TESS: Toronto Extremity Salvage Scale, 2MWT: 2-minute walk test, 6MWT: 6-minute walk test, 9MRWT: 9-minute run-walk test, CNS: central nervous system, CPET: cardio pulmonary exercise test, HR: heart rate, ECG: echocardiography, bpm: beats per minute, min: minute, km/h: kilometer per hour, PACER: progressive aerobic cardiovascular endurance run, s: second, W: watt, [1]only study participants who performed the assessments were counted, [2] if evaluated in a childhood cancer population

**Appendix 4:** Description of study methods assessing muscle strength in pediatric cancer patients and survivors

| Assessment | No. of studies/ total sample size[1] | Type of Cancer (No. participants) | Age range/ mean±SD [y] | Phases of treatment (No. studies) | Validity[2] | Reliability[2] | References |
|---|---|---|---|---|---|---|---|
| **Grip Strength Test** *measurement of maximum voluntary contraction of the hand using grip strength or hand dynamometer* | 27/4451 | Leukemia (2688)<br>Lymphoma (831)<br>CNS (538)<br>Neuroblastoma (40)<br>Retinoblastoma (11)<br>Renal tumor (35)<br>Bone tumor (29)<br>Soft tissue sarcoma (14)<br>Germ cell tumor (8)<br>Mixed (249)<br>*[sarcoma (2), hematologic malignancies (18), leukemia/lymphoma (59), bone/soft tissue sarcoma (13), neuroblastoma/Wilms tumor (49), other solid tumor (46), extracranial solid tumor (21), mixed malignancies (37)]*<br>Other (8)<br>*[Nasopharynx-CA (1), N/A (4), neuroectodermal tumor (3)]* | 3.5-64 | Active treatment (6)<br>Maintenance (1)<br>Off treatment (18)<br>Mixed (2)<br>*[active/off treatment (1), all phases (1)]* | No information | No information | Akyay et al. 2014; Bianco et al. 2014; Braam et al. 2018; Brinkman et al. 2018; Cox et al. 2018; DeFeo et al. 2020; Ehrhardt et al. 2017; Esbenshade et al. 2014; Gerber et al. 2006; Hartman et al. 2018; Hoffman et al. 2013; Howell et al. 2018; Järvelä et al. 2010; Joyce et al. 2011; Lam et al. 2018; Manchola-González et al. 2019; Ness et al. 2007; Ness et al. 2010; Ness DeLany et al. 2015; Ness, Kaste et al. 2015; Nielsen et al. 2018; Rosenhagen et al. 2011; Senn-Malashonak et al. 2019; Slater et al. 2015; Takken et al. 2009; Wright et al. 1998; Yildiz Kabak et al. 2016 |
| **Hand Held Dynamometry** *measurement of maximum voluntary isometric muscle strength using hand dynamometer* | 17/830 | Leukemia (489)<br>Lymphoma (29)<br>CNS tumor (182)<br>Neuroblastoma (2)<br>Renal tumor (28)<br>Bone tumor (17)<br>Soft tissue sarcoma (15)<br>Germ cell tumor (1)<br>Mixed (64)<br>*[ALL/Wilms tumor/other (6), solid tumor (16), mixed malignancies (25),* | 4-58 | Active treatment (6)<br>Maintenance (2)<br>Off treatment (6)<br>Mixed (3)<br>*[all phases (2), maint. /off treatmet (1)]* | No information | <u>Intra- and inter-rater reliability</u>: $r$ = 0.85-0.99 for knee extension and ankle dorsiflexion in 28 children with ALL aged 4-18y (Marchese, Chiarello & Lange, 2004) | Braam et al. 2016; Braam et al. 2018; Corr et al. 2017; Cox et al. 2018; Deisenroth et al. 2016; Esbenshade et al. 2014; Gilliam et al. 2011; Marchese et al. 2003; Hartman et al. 2008; Marchese, Chiarello & Lange 2004; Ness et al. 2007; Ness et al. 2010; Ness, Kaste et al. 2015; Oschwald et al. 2019; Takken et al. 2009; Tanner et al. 2015; van Brussel et al. 2006 |

| | | | | | | | |
|---|---|---|---|---|---|---|---|
| | | *lower-extremity sarcoma (14), N/A (3)]*<br>Other (3)<br>*[juvenile granulosa cell tumor) (1), malignant peripheral nerve sheath tumor (1), undifferentiated sarcoma of the liver (1)]* | | | | <u>Intra- and inter-rater reliability</u>:<br>ICC = 0.87-0.95 for knee extension and ankle dorsiflexion in 8 children with ALL aged 4-15y<br>( Marchese et al., 2003) | |
| **Isokinetic Dynamometry**<br>*measurement of maximum voluntary isokinetic muscle strength* | 10/3718 | Leukemia (2429)<br>Lymphoma (783)<br>CNS tumor (338)<br>Neuroblastoma (3)<br>Renal tumor (6)<br>Bone tumor (5)<br>Mixed (154)<br>*[leukemia/lymphoma (59), bone/soft tissue sarcoma (13), neuroblastoma/Wilms tumor (49), other solid tumor (30), sarcoma (3)]* | 10.5-64 | Maintenance (1)<br>Off treatment (9) | No information | <u>Test-retest reliability:</u><br>ICC = 0.86-0.99 in childhood cancer survivors and 0.71-0.97 in healthy controls for peak torque and total work done of trunk flexion and extension at 50°/s and 200°/s in **18 childhood cancer survivors and healthy controls aged 10-31y**<br>(Talvensaari et al., 1995) | Brinkman et al. 2018; DeFeo et al. 2020; Ehrhardt et al. 2017; Hoffman et al. 2013; Joyce et al. 2011; Muratt et al. 2011; Ness et al. 2012; Ness, DeLany et al. 2015; Smith et al. 2013; Talvensaari et al. 1995 |
| **Sit-Up Test**<br>*measurement of dynamic muscular endurance of trunk muscles* | 10/399 | Leukemia (165)<br>Lymphoma (8)<br>CNS tumor (39)<br>Neuroblastoma (32)<br>Retinoblastoma (11)<br>Renal tumor (34)<br>Bone tumor (1)<br>Soft tissue sarcoma (5) | 5-62.2 | Off treatment (9)<br>Mixed (1)<br>*[active/maint (1)]* | No information | No information | Bianco et al. 2014; Cortés-Reyes et al. 2013; Hartman et al. 2018; Hovi et al. 1993; Hovi et al. 2010; Howell et al. 2018; Järvelä et al. 2010; Long et al. 2017; Long et al. 2018; Taskinen et al. 2013 |

| | | | | | | | |
|---|---|---|---|---|---|---|---|
| | | Germ cell tumor (1)<br>Mixed (97)<br>*[hematologic malignancies (97)]*<br>Other (6) [N/A] | | | | | |
| **Push-Up Test** *measurement of dynamic muscular endurance of upper extremity; max reps or reps per 30s, 40s, or 60s counted* | 6/239 | Leukemia (102)<br>Lymphoma (8)<br>CNS tumor (39)<br>Neuroblastoma (32)<br>Retinoblastoma (11)<br>Renal tumor (34)<br>Bone tumor (1)<br>Soft tissue sarcoma (5)<br>Germ cell tumor (1)<br>Other (6) | 4-62.2 | Maintenance (1)<br>Off treatment (5) | No information | No information | Hartman et al. 2018; Hovi et al. 1993; Howell et al. 2018; Long et al. 2017; Long et al. 2018; Moyer-Mileur et al. 2009 |
| **Manual Muscle Test** *measurement of maximum voluntary isometric muscle strength using manual resistance and defined scales* | 6/165 | Leukemia (97)<br>Lymphoma (10)<br>Bone tumor (44)<br>Soft tissue sarcoma (3)<br>Mixed (8)<br>*[sarcoma (2), ALL/wilms tumor/other (6)]*<br>Other (3)<br>*[neuroectodermal (3)]* | 2-50+ | Active treatment (2)<br>Off treatment (2)<br>Mixed (2)<br>*[all phases (1); maint/off treatment(1)]* | No information | No information | Carty et al. 2009; Gerber et al. 2006; Nama et al. 2020; Schoenmakers et al. 2006; Tanner et al. 2015; Wright et al. 2017 |
| **Repetition Maximum (3RM/5RM/6RM)** *measurement of muscular endurance; maximum voluntary muscle strength during one or more repetitions of individual maximum resistance* | 6/104 | Leukemia (31)<br>Lymphoma (18)<br>CNS tumor (22)<br>Neuroblastoma (4)<br>Renal tumor (2)<br>Bone tumor (17)<br>Soft tissue sarcoma (7)<br>Germ cell tumor (1)<br>Other (2) *[N/A (2)]* | 4-23 | Active treatment (1)<br>Maintenance (2)<br>Off treatment (3) | No information | <u>Test-retest reliability:</u> ICC ≥ 0.977; ($p$ < 0.001) for seated bench press, leg press and seated lateral row in children with ALL aged 4-7 y; ICC ≥ 0.989 ($p$ < 0.001) for same tests in children with mixed cancers, mean age 10±1 y) (San Juan, Fleck, | Fiuza-Luces et al. 2017; Long et al. 2017; Long et al. 2018; San Juan et al. 2008; San Juan, Fleck, Chamorro-Viña, Maté-Muñoz et al. 2007; San Juan, Fleck, Chamorro-Viña, Moral et al. 2007 |

| | | | | | | | |
|---|---|---|---|---|---|---|---|
| | | | | | | Chamorro-Viña, Moral et al., 2007) | |
| **Repeated Squatting** *measurement of dynamic lower extremity muscular endurance* | 5/182 | Leukemia (79)<br>CNS tumor (22)<br>Mixed (79) [Hematologic malignancy (79)]<br>other (2) [N/A] | 6-30 | Off treatment (5) | No information | No information | Hovi et al. 2010; Järvelä et al. 2010; Long et al. 2017; Long et al. 2018; Taskinen et al. 2013 |
| **Back Extension Test** *measurement of dynamic or static muscular endurance* | 4/220 | Leukemia (87)<br>Neuroblastoma (26)<br>Renal tumor (28)<br>Mixed (79)<br>[Hematologic malignancy (79)] | 6-62.2 | Off treatment (4) | No information | No information | Hartman et al. 2018; Hovi et al. 2010; Järvelä et al. 2010; Taskinen et al. 2013 |
| **Isometric Dynamometry** *measurement of maximum voluntary isometric muscle strength, other than hand grip strength and hand-held dynamometry* | 3/143 | Leukemia (127)<br>Lymphoma (5)<br>Neuroblastoma (4)<br>Renal tumor (1)<br>Soft tissue sarcoma (5)<br>Other (1)<br>[*Nasopharynxcarcinoma (1)]* | 5-30 | Active treatment (1)<br>Off treatment (2) | No information | No information | Hovi et al. 1993; Senn-Malashonak et al. 2019; Tanir & Kuguoglu 2013) |
| **Chair-Stand Test** *measurement of dynamic lower extremity muscular endurance* | 3/100 | Leukemia (47)<br>Lymphoma (15)<br>CNS tumor (14)<br>Neuroblastoma (3)<br>Mixed solid tumors (21)<br>*[extracranial solid tumor (21)]* | 3.5-18 | Active treatment (2)<br>Off treatment (1) | No information | No information | Gilliam et al. 2011; Nielsen et al. 2018; Yildiz Kabak et al. 2016 |
| **Leg Lift Test** *measurement of dynamic muscular endurance of hip flexors* | 2/128 | Leukemia (49)<br>Mixed (79)<br>[Hematologic malignancy (79)] | 6-30 | Off treatment (2) | No information | No information | Hovi et al. 2010; Taskinen et al. 2013 |
| **Vertical Jump** *measurement of explosive muscle power of lower extremity* | 2/92 | Leukemia (38)<br>Neuroblastoma (26)<br>Renal tumor (28) | 16-62.2 | Off treatment (2) | No information | No information | Hartman et al. 2018; Järvelä et al. 2010 |
| **Shoulder Lift Test** | 1/21 | Leukemia (21) | 16-30 | Off treatment (1) | No information | No information | Järvelä et al. 2010 |

| | | | | | | | |
|---|---|---|---|---|---|---|---|
| *measurement of dynamic muscular endurance of upper extremity; max reps/side counted* | | | | | | | |
| **Standing Broad Jump** *measurement of explosive muscle power of lower extremity* | 1/18 | Hematologic malignancy (18) | 7.55±2.43 | Off treatment (1) | No information | No information | Bianco et al. 2014 |
| **Lateral Step Test** *measurement of dynamic lower extremity muscular endurance* | 1/12 | Leukemia (7)<br>CNS tumor (5) | 6-18 | Off treatment (1) | No information | No information | Gilliam et al. 2011 |

CNS: central nervous system, HHD: hand held dynamometer, RM: Repetition Maximum, reps: repetition, maint: maintenance treatment, No: Number, SD: standard deviation, [1]only study participants who performed the assessments were counted, [2] if evaluated in a childhood cancer population,

**Appendix 5:** Description of study methods assessing running speed in pediatric cancer patients and survivors

| Assessment | No. of studies/ total sample size[1] | Type of Cancer (No. participants) | Age range/ mean±SD [y] | Phases of treatment (No. studies) | Validity[2] | Reliability[2] | References |
|---|---|---|---|---|---|---|---|
| **Shuttle Run**<br>*Subject runs a shuttle run (10x5m or 4x 10m) as fast as possible; part of Eurofit Testing Battery* | 3/146 | Leukemia (49)<br>Mixed (97)<br>*[Hematologic malignancies (97)]* | 6-30 | Off treatment (3) | No information | No information | Hovi et al. 2010; Taskinen et al. 2013; Bianco et al. 2014 |
| **60m Run**<br>*Subjects runs 60m on a flat corridor/surface as fast as possible* | 2/105 | Leukemia (75)<br>Lymphoma (11)<br>CNS tumor (2)<br>Renal tumors (2)<br>Bone tumors (1)<br>Mixed (10)<br>*[Hematologic malignancies (10)]*<br>Other (4)<br>*[Nasopharynx-CA (1), Ovarian cancer (1), Evans syndrome (2)]* | 11±3 | Off treatment (2) | No information | No information | Malicka et al. 2019; Malicka et al. 2020 |

CNS: central nervous system, m: meter, maint: maintenance treatment, No: Number, SD: standard deviation, [1]only study participants who performed the assessments were counted, [2] if evaluated in a childhood cancer population

**Appendix 6:** Description of study methods assessing balance in pediatric cancer patients and survivors

| Assessment | No. of studies/ total sample size[1] | Type of Cancer (No. participants) | Age range/ mean±SD [y] | Phases of treatment (No. studies) | Validity[2] | Reliability[2] | References |
|---|---|---|---|---|---|---|---|
| Posturography | | | | | | | |
| **Balance tests (various) on force platforms:** *Ground reaction forces/center of pressure trajectory (COP sway) are recorded for 15s-60s at sampling frequencies of 50-60Hz. Measurements were performed under various conditions: i.e. quiet standing with eyes open/closed, Romberg test (heal-to-toe-stance) with eyes open/closed, standing on foam surface with eyes open/closed, single-leg stance (SLS)* | 4/292 | Leukemia (79)<br>CNS tumor (184)<br>Mixed (29)<br>[Bone and soft tissue sarcoma (29)] | 4-25.2 | Off treatment (3)<br>Mixed (1)<br>[during inpatient rehabilitation/off treatment and probably receiving maint. treatment (1)] | No information | No information | Galea et al. 2004;<br>Syczewska et al. 2008;<br>Syczewska et al. 2006;<br>Müller et al. 2017 |
| **Ultrasound-based motion analysis of postural sway (CM20, Zebris GmbH, Tübingen, Germany)** *during 10 different sensory conditions (sitting/standing on a regular surface eyes open/closed; sitting/standing on a cushion with eyes open/closed; standing in tandem position with eyes open/closed). An ultrasound receiver recorded pulses emitted by markes attached to the shoulders and trunk. Sampling frequency of the ultrasound system was 50 Hz. Testing of each condition was repeated four times. Each trial lasted 15 s.* | 1/22 | CNS tumor (22) | 11-39 | Off treatment (1) | No information | No information | Schoch et al., 2010 |
| **SOT (Sensory Organization Test) on a computerized dynamic posturography system (various):** *The SOT measures postural sway under six conditions: Conditions 1 to 3 have a fixed standing surface and are done with eyes open, eyes closed, and eyes open but sway referenced (visual surround moves in reference to anterior-posterior sway). Conditions 4 to 6 are the same but with a sway-referenced standing surface (the force plate moves in reference to* | 7/1.805 | Leukemia (780)<br>Lymphoma (200)<br>CNS tumor (350)<br>Mixed (475)<br>[Extracranial solid tumor (475)] | 10-63.8 | Off treatment (7) | No information | No information | Konczak et al. 2005;<br>Schoch et al. 2006;<br>Erhardt et al. 2017;<br>Ness et al. 2013;<br>Ness et al. 2012;<br>Ness et al. 2015;<br>Brinkmann et al. 2018 |

| | | | | | | | |
|---|---|---|---|---|---|---|---|
| *anterior-posterior sway). Scores from the six conditions are used to derive an overall SOT score. Two or three trials (20 seconds long) are collected for each condition.* | | | | | | | |
| **Non-Posturography** | | | | | | | |
| **The berg balance test:** *measures the ability to maintain an upright position during typical movements; 14 conditions are rated on a scale* | 1/156 | CNS tumor (156) | 18-58 | Off treatment (1) | No information | No information | Ness et al. 2010 |
| **Flamingo balance test:** The child is instructed to stand barefoot on one leg (preferred) for 60 sec. As the child loses balance, the timer is stopped and restarted once balance is regained. The number of restarts are recorded. | 1/75 | Leukemia (31)<br>Lymphoma (14)<br>CNS tumor (9)<br>Mixed (21)<br>*[extracranial solid tumor (21)]* | 11.3 ± 3.1 | Active treatment (1) | No information | No information | Nielsen et al. 2018 |
| **Single leg stance (SLS):** Child maintains SLS with hands on hips and eyes open for as long as possible. Time is measured in seconds. | 1/62 | Leukemia (62) | 1-22 | Active treatment (1) | No information | No information | Tanner et al. 2017 |

CNS: central nervous system, maint: maintenance treatment, No: Number, SD: standard deviation, SOT: sensory organization test; SLS: single leg stance, [1]only study participants who performed the assessments were counted, [2] if evaluated in a childhood cancer population

**Appendix 7:** Description of study methods assessing flexibility in pediatric cancer patients and survivors

| Assessment | No. of studies/ total sample size[1] | Type of Cancer (No. participants) | Age range/ mean±SD [y] | Phases of treatment (No. studies) | Validity[2] | Reliability[2] | References |
|---|---|---|---|---|---|---|---|
| **Goniometry:** *measurement of maximum active or passive joint motion using a Goniometer* | 33/3.749 | Leukemia (2649)<br>Lymphoma (811)<br>CNS (6)<br>Neuroblastoma (2)<br>Renal tumors (29)<br>Bone tumors (125)<br>Soft tissue sarcoma (17)<br>Germ cell tumor (2)<br>Mixed (101)<br>[sarcoma (2), solid non-CNS tumor (11), lower extremity sarcoma (82), no specific information (6)]<br>Other (7)<br>[neuroectodermal sarcoma (3), multiple diagnosis (1), juvenile granulosa cell tumor (1), malignant peripheral nerve sheath tumor (1), undifferentiated sarcoma of the liver (1)] | 1-64 | Active treatment (7)<br>Maintenance (4)<br>Off treatment (18)<br>Mixed (4)<br>[maint/off (3), all phases (1)] | No information | Intra-rater and inter-rater reliability for all measures ranged from r=0.85 to 0.99 in children with ALL aged 4-15y (Marchese et al. 2004)<br><br>Intrarater reliability coefficients were 0.760 for active and 0.927 for passive DF-ROM in children with ALL (Wright et al. 2003) | Beulertz et al. 2016; Carty et al. 2009; Ehrhardt et al. 2017; Esbenshade et al. 2014; Gerber et al. 2006; Gilchrist et al. 2016; Hartman et al. 2013; Hartman et al. 2008; Hartman et al. 2009; Henders et al. 2012; Tanir & Kuguoglu 2013; Marchese et al. 2004; Marchese et al. 2008; Marchese et al. 2006; Ness et al. 2014; Ness et al. 2015; Ness et al. 2012; Ness et al. 2015; San Juan et al. 2008; San Juan et al. 2008; San Juan et al. 2007; Tanner et al. 2015; Wright et al. 1999; Wright et al. 1998; Wright et al. 2003; Wurz et al. 2014; Corr et al. 2017; Cox et al. 2018; DeFeo et al. 2020; Oschwald et al. 2019; Tanner et al. 2018; Tanner et al. 2017; Wright et al. 2017 |
| **Sit and reach test:** *measurement of maximum hip flexion with legs extended while sitting using a sit and reach box* | 12/2.830 | Leukemia (1609)<br>Lymphoma (778)<br>CNS tumor (308)<br>Neuroblastoma (26)<br>Renal tumors (28)<br>Bone tumor (1)<br>Mixed (1)<br>[multiple diagnoses (1)] | 4-64 | Maintenance (2)<br>Off treatment (9)<br>Mixed (1)<br>[active/maint (1)] | No information | No information | Erhardt et al. 2017; Esbenshade et al. 2014; Moyer-Mileur et al. 2009; Ness et al. 2015; Taskinen et al. 2013; Wurz et al. 2014; Hovi et al. 2010; Brinkmann et al. 2018; DeFeo et al. 2020; Hartman et al. 2018; Manchola-Gonzales et al. 2019; Cortés-Reyes et al. 2013 |

| | | | | | | | |
|---|---|---|---|---|---|---|---|
| | | Other (79)<br>[hematological malign. (79)] | | | | | |
| **Side bending**: *Flexibility of the trunk was tested by side bending toward right and left. The difference in position of the middle finger on the lateral thigh was measured in upright position leaning against the wall with feet 15 cm apart and when side bending and the difference were expressed in centimeters.* | 1/71 | Leukemia (17)<br>Neuroblastoma (26)<br>Renal tumors (28) | 18.8-62.6 | Off treatment (1) | No information | No information | Hartman et al. 2018 |

CNS: central nervous system, maint: maintenance treatment, No: Number, SD: standard deviation, [1]only study participants who performed the assessments were counted, [2] if evaluated in a childhood cancer population

**Appendix 8:** Description of study methods assessing functional mobility in pediatric cancer patients and survivors

| Assessment | No. of studies/ total sample size[1] | Type of Cancer (No. participants) | Age range/ mean±SD [y] | Phases of treatment (No. studies) | Validity[2] | Reliability[2] | References |
|---|---|---|---|---|---|---|---|
| **TUG 3m:** *The Timed Up & Go test measures the time in seconds required to rise from sitting position in a standard arm chair, walk 3m/10feet, turn, walk back to the chair, and sit down.* | 25/4283 | Leukemia (2313)<br>Lymphoma (831)<br>CNS tumor (350)<br>Neuroblastoma (9)<br>Renal tumors (2)<br>Bone tumors (36)<br>Soft tissue sarcoma (9)<br>Germ cell tumor (2)<br>Mixed (728)<br>[without information (12), Leukemia or lymphoma (59), Bone or soft tissue sarcoma (13), Neuroblastoma or wilms tumor (49), extracranial solid tumor (496), other solid tumor (30), lower-extremity sarcoma (68) Multiple diagnosis (1)]<br>Other (3)<br>[(juvenile granulosa cell tumor) (1), malignant peripheral nerve sheath tumor (1), undifferentiated sarcoma of the liver (1)] | 3.5 – 64 | Active treatment (4)<br>Maintenance (2)<br>Off treatment (16)<br>Mixed (3)<br>[active/off (2), maint/off (1)] | **ALL patients during treatment:**<br>Correlation of r= 0.794 with knee extension strength<br>Correlation of r= -0.204 with ankle DF strength<br>*[Marchese et al. 2003]* | **ALL patients during treatment:**<br>r=0.99 [intra- and interrater ICC]<br>(Marchese et al. 2003)<br><br>**ALL patients during maintenance treatment:**<br>r=0.974 [Test-retest ICC]<br>(San Juan et al. 2007) | Ness et al 2013; Erhardt et al. 2017; Ness et al. 2007; San Juan et al. 2008; Akyay et al. 2014; San Juan et al. 2007; San Juan et al. 2007; Slater et al. 2015; Takken et al. 2009; Wurz et al. 2014; Kabak et al. 2016; Fiuza-Luces et al. 2016; Marchese et al. 2003; Hoffman et al. 2013; Marchese et al. 2004; Marchese et al. 2006; Ness et al. 2012; Brinkmann et al. 2018; DeFeo et al. 2020; Manchola-Gonzales et al., 2019; Nama et al. 2020; Nielsen et al. 2018; Oschwald et al. 2019; Varedi et al. 2019 |
| **TUDS:** *The TUDS Test requires an individual to ascend and descend 12 stairs while the time (in seconds) is recorded with a stopwatch. Participants begin this test from a* | 13/314 | Leukemia (175)<br>Lymphoma (19)<br>Neuroblastoma (7)<br>Renal tumors (2)<br>Bone tumors (35)<br>Soft tissue sarcoma (7)<br>Germ cell tumor (1)<br>Mixed (68)<br>[Lower-extremity sarcoma (68)] | 3.5 -27 | Active treatment (1)<br>Maintenance (4)<br>Off treatment (6)<br>Mixed (2)<br>[all phases (2)] | No information | **ALL patients during maintenance treatment:**<br>r=0.989 [Test-retest ICC]<br>(San Juan et al. 2007) | San Juan et al. 2008;<br>San Juan et al. 2008;<br>San Juan et al. 2007;<br>San Juan et al. 2007;<br>Takken et al. 2009;<br>Kabak et al. 2016;<br>Fiuza-Luces et al. 2016;<br>Marchese et al. 2004; Marchese et al. 2008; Marchese et al. 2004;<br>Marchese et al. 2006;<br>Manchola-Gonzales et al. 2019 |

| | | | | | | | |
|---|---|---|---|---|---|---|---|
| *standing position, with feet together and are instructed to go up and down the stairs as fast as possible while being safe. For safety reasons, participants are allowed to use the railing if desired* | | | | | | | |
| **TUG 10m:** *The timed up and go test is also conducted with a distance of 10m.* | 3/22 | Leukemia (22) | 4-16 | Maintenance (2)<br>Off treatment (1) | No information | **ALL patients during maintenance treatment:** r=0.997 [Test-retest ICC] (San Juan et al. 2007) | San Juan et al. 2008; San Juan et al. 2007; San Juan et al. 2007 |
| **Floor to stand performance**: *Rated floor to stand as independent or needs assistance.* | 1/62 | Leukemia (62) | 1-22 | Active treatment (1) | No information | No information | Tanner et al. 2017 |
| **Time needed to stand up from bed rest exam:** *subject instructed to stand up as quickly as possible from a lying position in bed to a standing position with hands at their sides using any independent method of their choice.* | 1/11 | Leukemia (7)<br>Lymphoma (1)<br>Neuroblastoma (3) | 3.5 - 15 | Active treatment (1) | No information | No information | Kabak et al. 2016 |

ALL: acute lymphoblastic leukemia, CNS: central nervous system, maint: maintenance treatment, No: Number, SD: standard deviation, TUDS: timed up and down stairs test, TUG: timed up and go test, [1]only study participants who performed the assessments were counted, [2] if evaluated in a childhood cancer population,

**Appendix 9:** Description of study methods assessing gait in pediatric cancer patients and survivors

| Assessment | No. of studies/ total sample size[1] | Type of Cancer (No. participants) | Age range/ mean±SD [y] | Phases of treatment (No. studies) | Validity[2] | Reliability[2] | References |
|---|---|---|---|---|---|---|---|
| **Video-recording, force plates and EMG measurements:** *Gait analysis was performed using 2-4 cameras (i.e. sampling at frequencies of 60Hz) and a set of reflective markers, as well as 2-3 force platforms and additional EMG-measurement (i.e. 14-16 channel surface EMG) in order to evaluate kinematic, kinetic and/or temporo-distal parameters. One study also calculated the Gait Deviation Index (GDI). Participants were asked to walk at self-selected speed on a defined walkway (8-12m) performing at least 6 trials.* | 3/67 | Leukemia (17)<br>Bone tumors (47)<br>Soft tissue sarcoma (2)<br>other (1)<br>[neuroectodermal tumor (1)] | 5-68.3 | Off treatment (2)<br>Mixed (1)<br>[maint/off treatment (1)] | No information | No information | Hillmann et al. 2001; Hillmann et al. 2000; Wright et al. 2017 |
| **Video-recording and force platforms*:*** *Gait analysis was performed using 6-8 cameras (i.e. sampling at frequencies between 20-100Hz) and a set of reflective markers, as well as 2-3 force platforms in order to evaluate kinematic, kinetic and/or temporo-distal parameters. Some studies also calculated i.e. Gait Deviation Index (GDI), Gillette Gait Index (GGI). Participants were asked to walk at self-selected/preferred/comfortable speed on a defined walkway (i.e. 6-9m) performing multiple (i.e. at least 5-6) trials.* | 6/111 | CNS tumor (41)<br>Bone tumors (57)<br>Germ cell tumor (13) | 3-35 | Off treatment (5)<br>Mixed (1)<br>[inclusion criteria said: at least one year post-surgery and completed adjuvant treatment program (without radiotherapy) (1)] | No information | No information | Fuchs et al. 2003; Syczewska et al. 2006; Zaccara et al. 2004; Carty et al, 2009; Benedetti et al. 2016; Pesenti et al. 2018 |
| **Video-recording:** *Gait analysis was performed using 2-8 cameras (i.e. sampling at frequencies between 50-100Hz) in order to evaluate kinematic and/or temporo-distal parameters. Some studies also calculated i.e. walk ratio, absolute symmetry index, Gillette Gait Index. Participants were asked to walk at self-selected speed (and in one study tandem* | 4/ 210 | CNS tumor (181)<br>Mixed (29)<br>[bone and soft tissue sarcoma (29)] | 4-24 | Off treatment (2)<br>Mixed (2)<br>[during inpatient rehabilitation (1), N/A (1-24 mon after surgery) (1)] | No information | No information | Bastian et al. 1998; Syczewska et al. 2010; Fiorillo et al. 2010; Müller et al. 2017 |

| | | | | | | | |
|---|---|---|---|---|---|---|---|
| *gait in addition) on a defined walkway (i.e. 6-10m) performing multiple (i.e. at least 5-6) trials.* | | | | | | | |
| **GaitRITE**<br>*14-ft portable GaitRITE electronic walkway, (rollup carpet containing electronic pressure sensors) (CIR Systems, Sparta, New Jersey)* | 2/58 | Leukemia (23)<br>Lymphoma (18)<br>Mixed (17)<br>[non-CNS solid tumor (11), ALL/Wilms/other (6)] | 5-22 | Active treatment (2) | No information | No information | Gilchrist et al. 2016; Tanner et al. 2015 |
| **Microgate Optogait 2D Gait analysis system**<br>*This system of optical detection is based on a transmitting and receiving bar each containing 96 LEDs per meter that communicate on an infrared frequency* | 1/13 | Leukemia (4)<br>Lymphoma (2)<br>CNS tumor (3)<br>Renal tumors (1)<br>Soft tissue sarcoma (2)<br>Germ cell tumor (1) | 6-15.8 | Off treatment (1) | No information | No information | Beulertz et al. 2016 |
| **Electromyographic analysis of gait**<br>*Walking on motor-driven treadmill, metal plates on shoe soles and conduction rubber of tread mill used to record gait ; 15minutes, speed of 0.83m/sec; mark was used for calculation of stride-time parameters and synchronisation of EMG and goniometric data; speed of 0.83-1.11m/sec for at least 3min for each speed* | 1/8 | Bone tumors (8) | N/A *(age at surgery: 5-19yrs and time since surgery 13-54 mo)* | Off treatment (1) | No information | No information | Steenhoff et al. 1993 |
| **10m Walk Test:** *The 10-meter walk test is an accepted method for measuring walking speed in the clinical setting and has already been applied in children with chronic diseases. It measures the time individuals need to walk 10-meters in either their preferred or their maximum walking speed. A marked walkway and a stopwatch were used in the current study as these are clinically feasible methods. Moreover, the 10-meter walk test was conducted on a straight path protocol with acceleration and deceleration phases outside* | 1/16 | Leukemia (4)<br>Lymphoma (3)<br>CNS tumor (1)<br>Neuroblastoma (2)<br>Soft tissue sarcoma (2)<br>Germ cell tumor (1)<br>other (3)<br>[(juvenile granulosa cell tumor) (1), malignant peripheral nerve sheath tumor (1), undifferentiated sarcoma of the liver (1))] | 6-19 | Mixed (1)<br>[maint/off (1)] | No information | No information | Oschwald et al. 2019 |

| | | | | | | | |
|---|---|---|---|---|---|---|---|
| *the timed portion in order to allow for a more precise measurement.* | | | | | | | |
| **Visual Observation:** *Observational analysis of one or more of the following descriptors as present or absent: normal, flatfoot strike, metatarsal strike, foot slap, toe drag, steppage gait, wide base, out-toeing, asymmetrical, and lateral lurch. These common gait abnormalities are assessed through visual observation* | 1/62 | Leukemia (62) | 1-22 | Active treatment (1) | No information | No information | Tanner et al. 2017 |

CNS: central nervous system, EMG: electromyography; m: meter, maint: maintenance treatment, No: Number, SD: standard deviation, [1]only study participants who performed the assessments were counted, [2] if evaluated in a childhood cancer population

**Appendix 10:** Description of assessments measuring motor performance with combined tests in pediatric cancer patients and survivors

| Assessment | No. of studies/ total sample size[1] | Type of Cancer (No. participants) | Age range/ mean±SD [y] | Phases of treatment (No. studies) | Validity[2] | Reliability[2] | References |
|---|---|---|---|---|---|---|---|
| **BOT-2 (Bruininks-Oseretsky Test of Motor Proficiency, 2nd edition, complete form):** Test battery with 8 subtests and 53 items: Subscale Fine manual control, 15 items (subtest 1: fine motor precision, subtest 2: fine motor integration); Subscale Manual coordination, 12 items (subtest 3: manual dexterity, subtest 7: upper-limb coordination); Subscale body coordination, 16 items (subtest 4: bilateral coordination, subtest 5: balance); Subscale strength & agility, 10 items (subtest 6: running speed and agility, subtest 8: strength) | 10/327 | Leukemia (127)<br>Lymphoma (43)<br>Leukemia/Lymphoma (45)<br>CNS tumor (86)<br>Renal tumors (7)<br>Bone tumors (2)<br>Soft tissue sarcoma (2)<br>Mixed (15)<br>[other solid tumor (15)] | 4-22 | Active treatment (4)<br>Off treatment (6) | No information | No information | Gilchrist et al. 2016*; Davis et al. 2010; Ramchandren et al. 2009; Piscione et al. 2014*; Sabel et al. 2015; Gilchrist & Tanner 2018*; Nama et al. 2019*; Piscione et al. 2017*; Tanner & Hooke 2019*; Tanner et al. 2017* |
| **BOT-2 SF (Bruininks-Oseretsky Test of Motor Proficiency, 2nd Edition, short form):** Test battery with 12 items consisting of at least one item from each BOT-2 subtest. | 6/384 | Leukemia (384) | 4-18 | Active treatment (1)<br>Maintenance (1)<br>Off treatment (3)<br>Mixed (1)<br>[active/maint. (1)] | No information | No information | Esbenshade et al. 2014; Ness et al. 2015; Hung et al. 2017; De Luca et al. 2013; Cox et al. 2018; Tay et al. 2017 |
| **m-ABC (Movement Assessment Battery for Children):** Test battery with eight tasks divided in three subsections: hand function, ball skills and balance skills | 5/283 | Leukemia (147)<br>Lymphoma (12)<br>Renal tumors (43)<br>Mixed (65)<br>Other (16) | 4.0-19.3 | Active treatment (2)<br>Off treatment (3) | No information | No information | Hartman et al. 2010; Hartman et al. 2009; Hartman et al., 2006; Reinders-Messelink et al. 1999; Kandula et al. 2018 |

| | | | | | | | |
|---|---|---|---|---|---|---|---|
| **BOT-MP (Bruininks-Oseretsky Test of Motor Proficiency):** Test battery with gross motor and fine motor composites; 8 subtests and 46 items<br><br>Gross motor composite:<br>Subtest 1: running speed & agility (1 item)<br>Subtest 2: balance (8 items)<br>Subtest 3: bilateral control (8 items)<br>Subtest 4: strength (3 items)<br>Subtest 5: Upper-limb coordination (9 items); Fine motor composite<br>Subtest 6: response speed (1 item)<br>Subtest 7: visual-motor control (8 items)<br>Subtest 8: upper limb speed and dexterity (8 items) | 5/164 | Leukemia (145)<br>Lymphoma (2)<br>Neuroblastoma (17) | 1.75-25.2 | Active treatment (1)<br>Maintenance (2)<br>Off treatment (2) | No information | No information | Wiernikowski et al. 2005*; Wright et al. 1998*; Mitchell et al. 2002*; Wright et al. 2005*; Tanner et al. 2017* |
| **m-ABC 2 (Movement Assessment Battery 2):** Test battery with 8 items in 3 dimensions: manual dexterity (3 items), aiming and catching (2 items) and balance (3 items) | 5/135 | Leukemia (113)<br>Lymphoma (14)<br>Renal tumors (3)<br>Bone tumors (1)<br>Soft tissue sarcoma (3)<br>Other solid tumor (1): [neoplasm of scrotum (1)] | 3-18.7 | Active treatment (1)<br>Off treatment (4) | No information | No information | Hartman et al. 2013; De Luca et al. 2013; Schoenmakers et al. 2006; Hamari et al. 2018; Oswald & Bo 2019 |
| **FMA (Functional Mobility Assessment):** Test battery with 6 subcategories: 1) pain (1 item); 2) function (2 items: TUDS, TUG), 3) supports; 4) | 4/276 | Bone tumors (274)<br>Soft tissue sarcoma (2) | 10.4-42.4 | Active treatment (1)<br>Off treatment (3) | No information | No information | Ness et al. 2014; Ginsberg et al. 2007; Corr et al. 2017; Marchese et al. 2007 |

| | | | | | | | |
|---|---|---|---|---|---|---|---|
| satisfaction with walking quality; 5) participation in work, school, sports; 6) endurance (9min walk/run) | | | | | | | |
| **MOON-test (test for motor performance in pediatric oncology):** Test battery for children with cancer with 8 items for strength (hand grip strength, sit to stand, medicine ball shot), speed (reaction test), coordination (single leg stance, inserting pins, throwing at target), and flexibility (stand and reach) | 4/141 | Leukemia (44)<br>Lymphoma (14)<br>CNS tumor (4)<br>Renal tumors (5)<br>Bone tumors (62)<br>Soft tissue sarcoma (6)<br>Germ cell tumor (1)<br>Mixed (5)<br>[other solid tumor (5)] | 4-23 | Active treatment (2)<br>Off treatment (1)<br>Mixed (1)<br>[all phases (1)] | No information | No information | Kesting et al. 2015; Götte et al. 2015; Götte & Kesting et al. 2013; Götte & Kesting et al. 2018 |
| **DMT 6-18 (motor performance test for 6-18 years olds:** Test battery with 8 items, for endurance (6 min run), strength (20m sprint, push-up, sit-up, standing long jump), coordination under time pressure (sideways jumps), coordination under precision pressure (balancing backwards), and flexibility (forward bend) | 4/70 | Leukemia (25)<br>Lymphoma (9)<br>CNS tumor (13)<br>Renal tumors (3)<br>Bone tumors (2)<br>Soft tissue sarcoma (8)<br>Germ cell tumor (6)<br>Other (4) | 6-17 | Off treatment (2)<br>Mixed (2) [maint./off (2)] | No information | No information | Beulertz et al. 2016, Beulertz et al. 2016, Beulertz et al. 2013; Däggelmann et al. 2017 |
| **GMFM (Gross motor function measure):** Test battery to measure change in gross motor function over time (cerebral palsy). Items span the spectrum of gross motor activities in five dimensions. A: Lying and Rolling, B: Sitting, C: Crawling and | 4/62 | Leukemia (60)<br>Lymphoma (2) | 2-14.6 | Active treatment (2)<br>Maintenance (1)<br>Off treatment (1) | Construct validity: paired T-tests demonstrated significant, positive score changes during a period of | Inter-rater Rel: D: 0.99; E: 0.99<br>Test-retest Rel: D: 0.97; E: 0.96<br>(Wright et al. 2007) | Wiernikowski et al. 2005*; Wright et al., 1998*; Gohar et al., 2011; Cortes-Reyes et al. 2013* |

| | | | | | | | |
|---|---|---|---|---|---|---|---|
| Kneeling, D: Standing, and E: Walking, Running and Jumping. | | | | | clinically observed change: D: t=6.40, p<0.001 E: t=7.31, p<0.001 (Wright et al. 2007) | | |
| **MOT 4-6 (motor performance test for 4-6 years olds):** Test battery with 18 items covering motor capabilities: agility and coordination, fine motor function, balance, ability to react, jumping power, motor speed, and motor control | 3/22 | Leukemia (7) Lymphoma (5) Neuroblastoma (2) Germ cell tumor (3) Other (5) | 3.42-5.42 | Off treatment (1) Mixed (2) [maint/off treatment (2)] | No information | No information | Beulertz et al. 2016; Beulertz et al. 2013; Däggelmann et al. 2017 |
| **Lincoln-Oseretzky Motor Development Scale**: Test battery (Motor Development Scale) with 36 items involving a variety of motor skills such as finger dexterity, eye-hand coordination, and gross activity of hands, arms, legs, and trunk. | 1/45 | Leukemia (31) Mixed (14) | 5-14 | Off treatment (1) | No information | No information | Harten et al. 1984 |
| **FMS - fundamental movement skills test battery**: Test battery with 7 physical movements: 4 locomotor skills: sprint run, vertical jump, side gallop and leaping; and 3 object control (or manipulative) skills: throwing, catching, and kicking | 1/26 | Leukemia (17) Lymphoma (1) CNS tumor (4) Renal tumors (3) Other (1) *[Sarcoma (1)]* | 5-8 | Off treatment (1) | No information | No information | Naumann et al. 2015 |
| **GMFM-ALL (Gross motor function measure ALL):** Test | 1/20 | Leukemia (20) | 2.8-15.9 | Mixed (1) [active/ maint. treatment (1)] | Construct validity: | Inter-rater-Rel: D and E: 0.99 | Wright et al. 2007 |

| | | | | | | | |
|---|---|---|---|---|---|---|---|
| battery for children with leukemia (selection of significant items of GMFM dimensions D - standing (7 items) and E – walking, running, jumping (13 items) | | | | | paired T-tests demonstrated significant, positive score changes during a period of clinically observed change: D: t=6.86, p<0.001 E: t=6.85, p<0.001 (Wright et al. 2007) | Test-retest Rel: D: 0.95 and E: 0.94 (Wright et al. 2007) | |
| **UQAC-UQAM Test Battery:** Test battery with 5 main factors, which are composed of 11 different tests. 1) Agility tests; 2) Coordination; 3) Balance; 4) Simple reaction test; 5) Limb speed | 1/20 | Leukemia (20) | 9-11 | Off treatment (1) | Jackknife validation (correct classification cases): 88.4% | No information | Leone et al. 2014 |
| **Physical fitness battery test, adapted by the ALPHA-fitness test battery**: Test battery with standing broad jump, hand grip test, the 4 x 10 m shuttle run test and sit-up test | 1/18 | Leukemia/Lymphoma (18) | 7.55 ± 2.43 | Off treatment (1) | No information | No information | Bianco et al. 2014 |
| **FITNESSGRAM®**: Test battery for health related fitness; state, district, school, teacher, and individual reporting; student cognitive tests | 1/10 | Leukemia (4) Lymphoma (4) CNS tumor (1) Germ cell tumor (1) | 14.0-18.0 | Mixed (1) [active/off treatment (1)] | No information | No information | Keats et al. 2008* |

BOT: Bruininks-Oseretsky Test, SF: short form; m-ABC: Movement Assessment Battery for Children, BOTMP: Bruininks-Oseretsky Test of Motor Proficiency, FMA: Functional Mobility Assessment, MOON: Motor Performance in Pediatric Oncology, DMT: Deutscher Motorik Test, GMFM: Gross motor function measure, MOT: Motoriktest für Kinder, FMS: fundamental movement skills test battery, ALL: acute lymphoblastic leukemia, UQAC-UQAM: University of Québec in Chicoutimi-University of Québec in Montréal, CNS: central nervous system, maint.: maintenance treatment, No: Number, SD: standard deviation

[1]only study participants who performed the assessments were counted, [2] if evaluated in a childhood cancer population, * Those studies only used subtests of the motor performance battery

# PRISMA 2009 Checklist

| Section/topic | # | Checklist item | Reported on page # |
|---|---|---|---|
| **TITLE** | | | |
| Title | 1 | Identify the report as a systematic review, meta-analysis, or both. | 1 |
| **ABSTRACT** | | | |
| Structured summary | 2 | Provide a structured summary including, as applicable: background; objectives; data sources; study eligibility criteria, participants, and interventions; study appraisal and synthesis methods; results; limitations; conclusions and implications of key findings; systematic review registration number. | 2 |
| **INTRODUCTION** | | | |
| Rationale | 3 | Describe the rationale for the review in the context of what is already known. | 3 |
| Objectives | 4 | Provide an explicit statement of questions being addressed with reference to participants, interventions, comparisons, outcomes, and study design (PICOS). | 4 |
| **METHODS** | | | |
| Protocol and registration | 5 | Indicate if a review protocol exists, if and where it can be accessed (e.g., Web address), and, if available, provide registration information including registration number.<br>→ Not registered because no direct health outcome was evaluated. | N/A |
| Eligibility criteria | 6 | Specify study characteristics (e.g., PICOS, length of follow-up) and report characteristics (e.g., years considered, language, publication status) used as criteria for eligibility, giving rationale. | 4-5 |
| Information sources | 7 | Describe all information sources (e.g., databases with dates of coverage, contact with study authors to identify additional studies) in the search and date last searched. | 4 |
| Search | 8 | Present full electronic search strategy for at least one database, including any limits used, such that it could be repeated. | Appendix 1 |
| Study selection | 9 | State the process for selecting studies (i.e., screening, eligibility, included in systematic review, and, if applicable, included in the meta-analysis). | 5 |
| Data collection process | 10 | Describe method of data extraction from reports (e.g., piloted forms, independently, in duplicate) and any processes for obtaining and confirming data from investigators. | 5-6 |
| Data items | 11 | List and define all variables for which data were sought (e.g., PICOS, funding sources) and any assumptions and simplifications made. | 5-6 |
| Risk of bias in individual studies | 12 | Describe methods used for assessing risk of bias of individual studies (including specification of whether this was done at the study or outcome level), and how this information is to be used in any data synthesis.<br>→ Not applicable for assessment summary | N/A |
| Summary measures | 13 | State the principal summary measures (e.g., risk ratio, difference in means). | 4 |

| Section/topic | # | Checklist item | Reported on page # |
|---|---|---|---|
| Synthesis of results | 14 | Describe the methods of handling data and combining results of studies, if done, including measures of consistency (e.g., $I^2$) for each meta-analysis. | N/A |



| Section/topic | # | Checklist item | Reported on page # |
|---|---|---|---|
| Risk of bias across studies | 15 | Specify any assessment of risk of bias that may affect the cumulative evidence (e.g., publication bias, selective reporting within studies). | N/A |
| Additional analyses | 16 | Describe methods of additional analyses (e.g., sensitivity or subgroup analyses, meta-regression), if done, indicating which were pre-specified. | N/A |
| **RESULTS** | | | |
| Study selection | 17 | Give numbers of studies screened, assessed for eligibility, and included in the review, with reasons for exclusions at each stage, ideally with a flow diagram. | 6 |
| Study characteristics | 18 | For each study, present characteristics for which data were extracted (e.g., study size, PICOS, follow-up period) and provide the citations. | Appendix 2-10 |
| Risk of bias within studies | 19 | Present data on risk of bias of each study and, if available, any outcome level assessment (see item 12). | N/A |
| Results of individual studies | 20 | For all outcomes considered (benefits or harms), present, for each study: (a) simple summary data for each intervention group (b) effect estimates and confidence intervals, ideally with a forest plot. | N/A |
| Synthesis of results | 21 | Present the main results of the review. If meta-analyses are done, include for each, confidence intervals and measures of consistency. | 7-11 |
| Risk of bias across studies | 22 | Present results of any assessment of risk of bias across studies (see Item 15). | N/A |
| Additional analysis | 23 | Give results of additional analyses, if done (e.g., sensitivity or subgroup analyses, meta-regression [see Item 16]). | N/A |
| **DISCUSSION** | | | |
| Summary of evidence | 24 | Summarize the main findings including the strength of evidence for each main outcome; consider their relevance to key groups (e.g., healthcare providers, users, and policy makers). | 11 |
| Limitations | 25 | Discuss limitations at study and outcome level (e.g., risk of bias), and at review-level (e.g., incomplete retrieval of identified research, reporting bias). | 13/14 |
| Conclusions | 26 | Provide a general interpretation of the results in the context of other evidence, and implications for future research. | 12/13 |
| **FUNDING** | | | |
| Funding | 27 | Describe sources of funding for the systematic review and other support (e.g., supply of data); role of funders for the systematic review. | 1 |

*From:* Moher D, Liberati A, Tetzlaff J, Altman DG, The PRISMA Group (2009). Preferred Reporting Items for Systematic Reviews and Meta-Analyses: The PRISMA Statement. PLoS Med 6(7): e1000097. doi:10.1371/journal.pmed1000097
